# Genetic regulation of methylation in human endometrium and blood and gene targets for reproductive diseases

**DOI:** 10.1186/s13148-019-0648-7

**Published:** 2019-03-14

**Authors:** Sally Mortlock, Restuadi Restuadi, Rupert Levien, Jane E. Girling, Sarah J. Holdsworth-Carson, Martin Healey, Zhihong Zhu, Ting Qi, Yang Wu, Samuel W. Lukowski, Peter A. W. Rogers, Jian Yang, Allan F. McRae, Jenny N. Fung, Grant W. Montgomery

**Affiliations:** 1 Institute for Molecular Bioscience, The University of Queensland, 306 Carmody Road, Building 80, St Lucia, QLD 4072 Australia; 2 Department of Obstetrics and Gynaecology, and Gynaecology Research Centre, University of Melbourne, Royal Women’s Hospital, Parkville, VIC 3052 Australia; 3 Department of Anatomy, University of Otago, Dunedin, New Zealand

**Keywords:** DNA methylation, DNA methylation quantitative trait loci (mQTL), Endometrium, Blood, Menstrual cycle, Endometriosis

## Abstract

**Background:**

Major challenges in understanding the functional consequences of genetic risk factors for human disease are which tissues and cell types are affected and the limited availability of suitable tissue. The aim of this study was to evaluate tissue-specific genotype-epigenetic characteristics in DNA samples from both endometrium and blood collected from women at different stages of the menstrual cycle and relate results to genetic risk factors for reproductive traits and diseases.

**Results:**

We analysed DNA methylation (DNAm) data from endometrium and blood samples from 66 European women. Methylation profiles were compared between stages of the menstrual cycle, and changes in methylation overlaid with changes in transcription and genotypes. We observed large changes in methylation (27,262 DNAm probes) across the menstrual cycle in endometrium that were not observed in blood. Individual genotype data was tested for association with methylation at 443,016 and 443,101 DNAm probes in endometrium and blood respectively to identify methylation quantitative trait loci (mQTLs). A total of 4546 sentinel *cis*-mQTLs (*P* < 1.13 × 10^−10^) and 434 sentinel *trans*-mQTLs (*P* < 2.29 × 10^−12^) were detected in endometrium and 6615 sentinel *cis*-mQTLs (*P* < 1.13 × 10^−10^) and 590 sentinel *trans*-mQTLs (*P* < 2.29 × 10^−12^) were detected in blood. Following secondary analyses, conducted to test for overlap between mQTLs in the two tissues, we found that 62% of endometrial *cis*-mQTLs were also observed in blood and the genetic effects between tissues were highly correlated. A number of mQTL SNPs were associated with reproductive traits and diseases, including one mQTL located in a known risk region for endometriosis (near *GREB1*).

**Conclusions:**

We report novel findings characterising genetic regulation of methylation in endometrium and the association of endometrial mQTLs with endometriosis risk and other reproductive traits and diseases. The high correlation of genetic effects between tissues highlights the potential to exploit the power of large mQTL datasets in endometrial research and identify target genes for functional studies. However, tissue-specific methylation profiles and genetic effects also highlight the importance of also using disease-relevant tissues when investigating molecular mechanisms of disease risk.

**Electronic supplementary material:**

The online version of this article (10.1186/s13148-019-0648-7) contains supplementary material, which is available to authorized users.

## Background

Genetic risk factors for complex disease mostly reside in non-coding regions of the genome [1, 2] and studies integrating results from genome-wide association studies and the genetic effects on methylation and gene expression provide a powerful approach to understand the functional consequences of these genetic risk factors. DNA methylation (DNAm) is one of the most common forms of epigenetic modification and involves the addition of a methyl group to the carbon-5 position of cytosine, often occurring at CpG sites [3]. Methylation is essential in facilitating embryonic development, chromosomal infrastructure, cell viability, imprinting, X chromosome-inactivation and transcription [3–6]. Methylation patterns in DNA samples from blood are associated with disease pathogenesis and are influenced by underlying genetic variation [7–10]. Difficulty accessing disease-relevant tissues has meant many studies make use of large gene expression and methylation datasets from peripheral blood as a proxy. However, differences in methylation profiles contribute to tissue-specific functions [11–13] and understanding tissue specificity of methylation signals is important to help interpret the role of methylation in disease risk.

The human endometrium is a highly specialised tissue lining the inside of the uterus and is essential to implantation, development of the placenta, and successful pregnancy [14]. Endometrium undergoes a cyclic process of cellular proliferation, differentiation, degradation, and regeneration [14, 15]. This dynamic process is accompanied by marked changes in gene expression that occur in response to changes in circulating concentrations of the steroid hormones oestradiol and progesterone [14, 16–18]. Methylation profiles in human endometrium also change across the menstrual cycle with thousands of genes differentially methylated between cycle stages [19–22].

This study aimed to compare DNA methylation patterns in both endometrium and blood collected from women sampled at different stages across the menstrual cycle. We identified methylation quantitative trait loci (mQTLs) in endometrium and correlated the mQTLs with blood mQTLs in the same women, and with larger mQTL datasets. We then evaluated overlap of mQTLs in endometrium with oestrogen receptor (ESR) binding sites and the overlap of mQTLs in both tissues with genomic regions associated with risk for endometriosis and other reproductive disorders. Results from this study provide novel insight into genetic control of methylation in human endometrium through the identification of endometrial mQTLs. Our work highlights methylation differences between blood and endometrial tissues across the menstrual cycle, and similarities between blood and endometrium in genetic regulation of methylation.

## Results

### Genome-wide methylation profiles

**Fig. 1:**
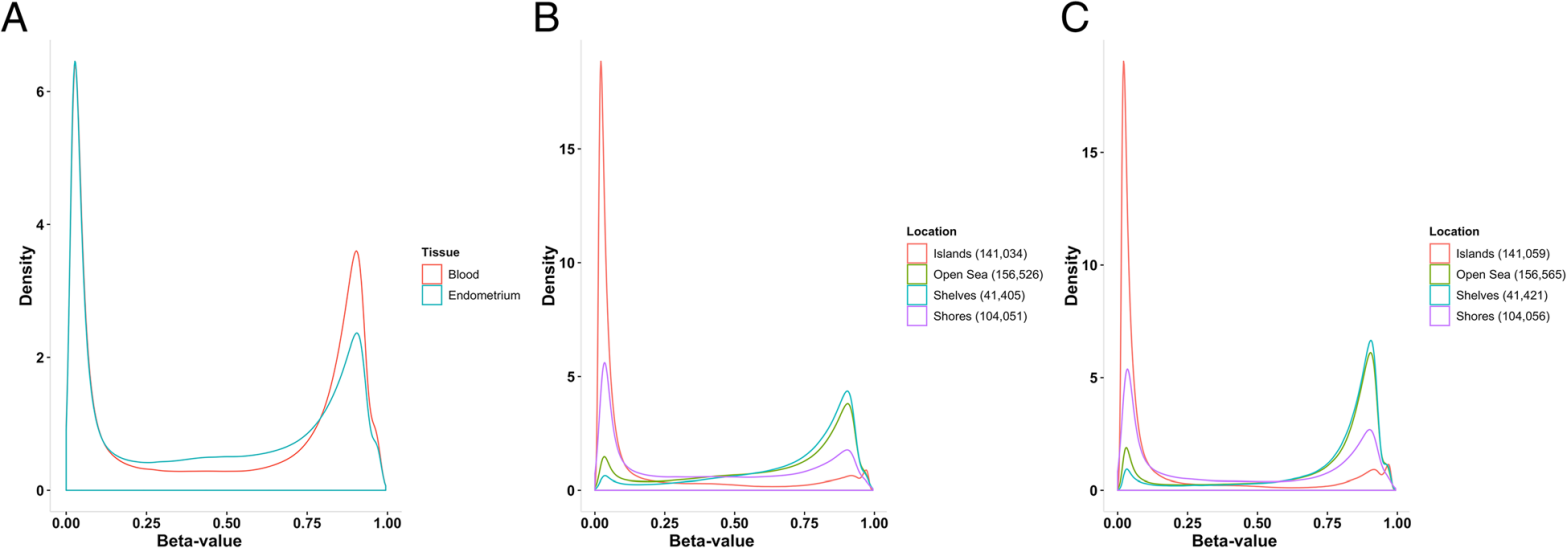
**a** Density plot showing the distribution of beta values measured at each DNA methylation (DNAm) probe in endometrium and blood. **b** Density plot showing the distribution of beta values measured at each DNAm probe in endometrium, values grouped according to location to CPG islands. **c** Density plot showing the distribution of beta values measured at each DNAm probe in blood, values grouped according to location to CPG islands

We analysed genome-wide methylation profiles in endometrium from 66 European women who attended clinics at the Royal Women’s Hospital in Melbourne, Australia. Following quality control (QC) filtering, a total of 443,016 and 443,101 DNAm probes remained for analyses in endometrial tissue samples and blood samples, respectively. Both endometrium and blood had a similar proportion of probes sites consistently hypomethylated (Fig. 1a). However, a larger proportion of probes sites in blood were consistently hypermethylated (Fig. 1a) (Additional file 1: Supplementary Note 1). CpG probe sites were annotated according to their proximity to CpG islands using the Illumina Human Methylation 450 BeadChip manifest file (see Additional file 1: Supplementary Note 1 for definitions and detailed results). Hypomethylated sites were more common in CpG islands and hypermethylated sites were more common in open sea regions in both endometrium and blood (Fig. 1b, c).

**Fig. 2:**
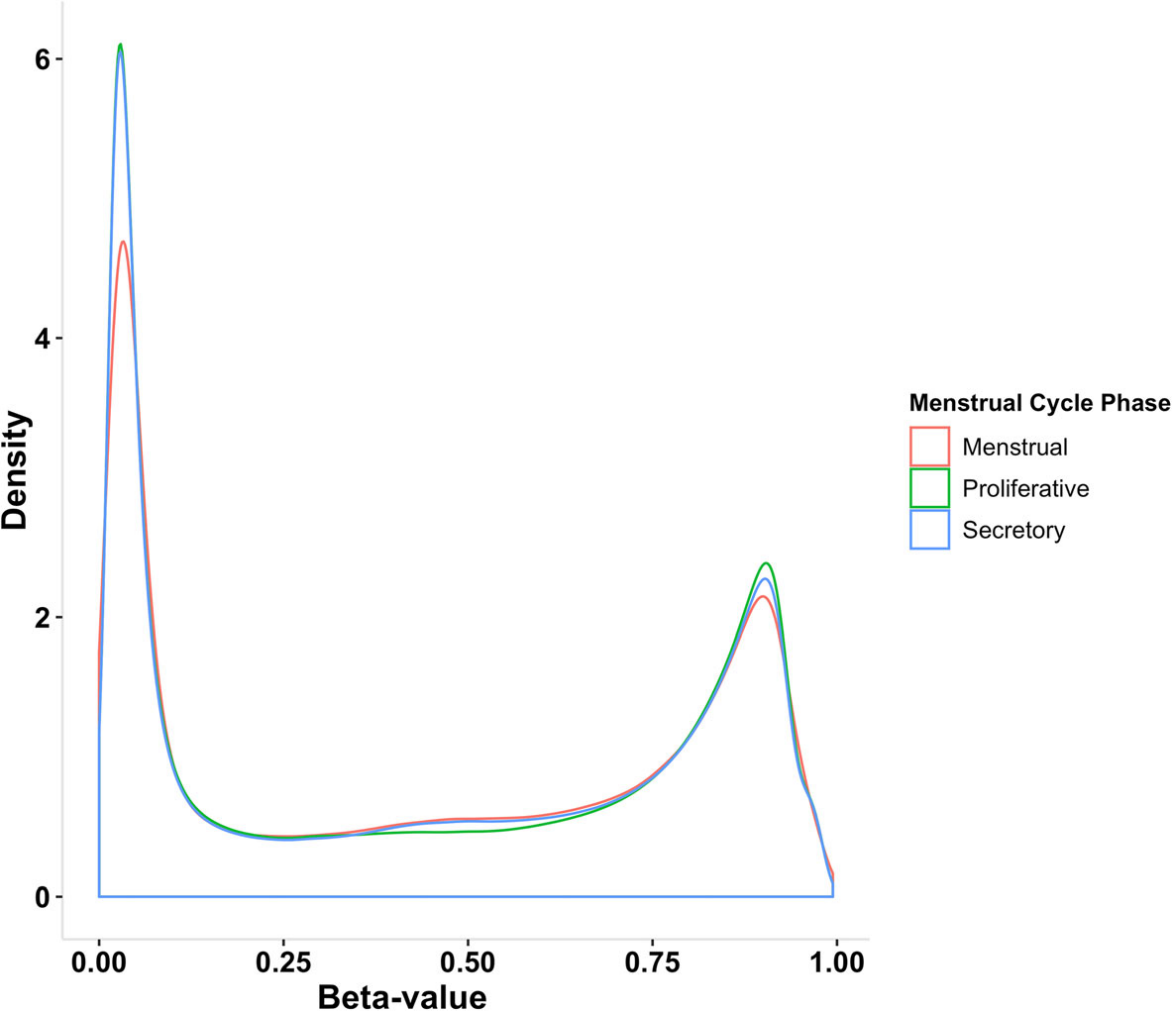
Density plot showing the distribution of beta values measured at each DNA methylation (DNAm) probe in endometrium from three menstrual cycle hases, the menstrual phase, proliferative phase and secretory phase

We see very similar genome-wide methylation profiles between menstrual (M), proliferative (P) and secretory (S) phases of menstrual cycle in endometrium. In all cycle phases, we observed 35.2–36% of probes consistently hypomethylated in at least 90% of individuals and 22.6–23.9% of probes consistently hypermethylated in individuals (Fig. 2). We also see similar methylation patterns for probes located in CpG islands, shores, shelves and open sea regions across all three phases (Additional file 2: Figure S1).

### Differential DNA methylation

To investigate changes in methylation across the menstrual cycle in endometrium and blood, we performed a differential methylation analysis between the proliferative (P) phase and secretory (S) phase of menstrual cycle. Stage of cycle was determined by histological assessment of endometrial tissue. We detected significant differences in methylation in endometrium for 6% of DNAm probe sites (*n =* 27,262) across the menstrual cycle comparing the P phase and S phases of the cycle (FDR < 0.05, *P* < 3.07 × 10^−3^) (Additional file 3: Table S1). Each DNAm probe site was annotated to the gene with the closest transcription start site (11,281 genes). The top 30 differentially methylated DNAm probe sites between the two phases of the menstrual cycle in endometrial tissue are listed in Table 1 and shown in Additional file 2: Figure S2. Marked changes in the methylation of 40 DNAm probe sites with the largest fold change between the proliferative and secretory phase are shown in Additional file 2: Figure S3. The majority of differentially methylated DNAm probe sites (51.9%) were concentrated in open sea locations and CpG island shores (25.3%) throughout the genome (Additional file 3: Table S2, Additional file 2: Figure S4). Differences observed across the cycle in endometrial tissue were not reflected in blood and are likely, in part, to reflect changes in cell composition.

**Table 1:** Differentially methylated DNAm probe sites in endometrium. The top 30 significantly differentially methylated DNAm probe sites between the proliferative (P) and secretory (S) phase (PvsS)

DNAm probe site ID	Log2 fold change	*P* value	Adjusted *P* value	Probe start (hg19)	Closest TSS gene name
cg16201273	0.146	7.96E-15	3.53E-09	80,855,803	*ZMIZ1*
cg20888995	0.146	2.74E-11	6.06E-06	56,822,059	*ARHGEF3*
cg01713086	0.128	1.44E-10	1.60E-05	28,268,413	*ZNF395*
cg07730183	0.096	1.45E-10	1.60E-05	2,136,400	*TSC2*
cg22934449	0.093	4.52E-10	3.72E-05	104,199,723	*ZFYVE21*
cg21369890	0.137	5.28E-10	3.72E-05	86,099,963	*AK024998*
cg06669056	0.096	6.17E-10	3.72E-05	5,570,715	*C4orf6*
cg25237396	− 0.092	6.71E-10	3.72E-05	802,148	*MIR4745*
cg17900103	0.114	1.45E-09	6.33E-05	20,940,931	*PINK1*
cg22185879	− 0.087	1.53E-09	6.33E-05	62,153,192	*PPDPF*
cg23235622	0.096	1.57E-09	6.33E-05	34,039,299	*CEP250*
cg25735294	0.103	2.99E-09	1.10E-04	186,353,671	*FETUB*
cg02248729	− 0.047	4.76E-09	1.62E-04	80,555,018	*FOXK2*
cg12082793	0.097	6.48E-09	2.05E-04	20,218,923	*OTUD3*
cg11224737	0.133	8.02E-09	2.37E-04	72,991,072	*LOC728978*
cg26479868	0.082	1.00E-08	2.77E-04	29,916,194	*TMTC1*
cg09714100	− 0.092	1.31E-08	3.42E-04	44,821,342	*SIK1*
cg27133780	0.133	1.41E-08	3.46E-04	32,474,743	*CMTM7*
cg21163015	0.108	1.82E-08	4.25E-04	140,658,386	*FLJ40292*
cg02118194	− 0.040	2.37E-08	5.09E-04	46,404,488	*MYPOP*
cg03018949	0.062	2.68E-08	5.09E-04	127,371,608	*C10orf122*
cg26469099	0.085	2.78E-08	5.09E-04	4,144,866	*PARP11*
cg22182975	0.071	2.86E-08	5.09E-04	167,571,122	*GPR31*
cg05224671	0.090	3.15E-08	5.09E-04	65,435,408	*RAB15*
cg09616559	− 0.066	3.22E-08	5.09E-04	25,921,150	*Y*_*RNA*
cg22416376	0.092	3.33E-08	5.09E-04	17,395,271	*SLC7A2*
cg09100343	0.075	3.34E-08	5.09E-04	57,147,152	*CPNE2*
cg25420952	0.084	3.46E-08	5.09E-04	116,841,084	*AMBP*
cg18645625	0.102	3.48E-08	5.09E-04	79,699,531	*ZFYVE16*
cg21642947	− 0.062	3.83E-08	5.09E-04	62,153,431	*PPDPF*

Gene lists corresponding to the closest transcription start sites (TSS) to differentially methylated DNAm probe sites in endometrial tissue across the cycle were compared to genes found to be differentially expressed between the same stages in endometrial tissue using data from Fung et al. [17]. Over a quarter of genes annotated to differentially methylated sites (3215 genes) were also differentially expressed between the proliferative and secretory phases (Additional file 2: Figure S5). This overlap with differentially expressed genes was significantly different to the proportion expected by chance (chi-square statistic = 5.10, *P* = 0.02).

### mQTL analysis

**Fig. 3:**
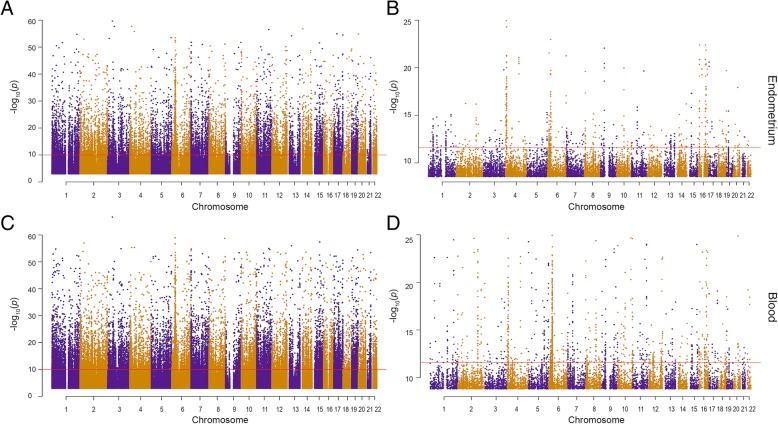
Manhattan plots of endometrial tissue (top; panels **a**, **b**) and blood (bottom; panels **c**, **d**) *cis* (left; panels **a**, **c**) and *trans*-mQTLs (right; panels **b**, **d**). Each point represents an mSNP, chromosomes are defined by alternating purple and orange points and the red line indicates a Bonferroni threshold of *P* < 1.13 × 10^−10^ for *cis*-eQTLs and *P* < 2.29 × 10^−12^ for *trans*-eQTLs

Using genotype information from each individual, we performed an expression quantitative trait loci (eQTL) analysis to identify associations between SNPs and DNAm probes in both endometrial tissue and blood. In endometrial tissue, we observed 4546 sentinel *cis*-mQTLs (*P* < 1.13 × 10^−10^) and 434 sentinel *trans*-mQTLs (*P* < 2.29 × 10^−12^), using a Bonferroni threshold to correct for multiple testing. Sentinel *cis*-mQTLs were defined as the mQTL with the most significant *P* value for each DNAm probe and sentinel *trans*-mQTLs were defined as mQTLs harbouring independent (R^2^ < 0.5) SNPs on a different chromosome to the associated DNAm site. There were similar numbers in blood DNA with 6615 sentinel *cis*-mQTLs (*P* < 1.13 × 10^−10^) and 590 sentinel *trans*-mQTLs (*P* < 2.29 × 10^−12^) (Fig. 3). The 30 most significant *cis*-mQTLs identified in endometrial tissue are listed in Table 2 and the 30 most significant *cis*-mQTLs identified in blood are listed in Table 3. Conditional analysis on *cis*-mQTLs reaching Bonferroni significance identified secondary *cis*-mQTL signals for 9 DNAm probe sites in endometrial tissue and 44 DNAm probe sites in blood. Only 23 DNAm probe sites were both differentially methylated across the cycle and had a *cis*-mQTL in endometrial tissue. We found no interaction between genotype and stage of cycle at these 23 DNAm probe sites.

**Table 2:** Top 30 most significant *cis*-mQTLs in endometrium

CHR	SNP	BP	A1	BETA	SE	*P* value	DNAm probe site ID	Probe start (hg19)	Closest TSS gene name
3	rs6783741	43,455,926	T	0.4525	0.00261	2.23E-60	cg11035303	43,465,453	*SNRK*
4	rs5856334	16,076,153	AT	0.3958	0.002547	1.94E-58	cg17858192	16,077,757	*PROM1*
3	3:61237223:T:C	61,237,223	T	0.4387	0.002831	2.20E-58	cg17573813	61,237,223	*FHIT*
14	rs12436555	24,634,825	A	− 0.4469	0.003038	1.85E-57	cg02898977	24,662,177	*IPO4*
11	rs73555593	107,462,942	A	0.4494	0.003092	3.00E-57	cg22355889	107,461,585	*ELMOD1*
4	rs6854452	39,446,337	A	0.4824	0.003458	1.62E-56	cg19311470	39,460,490	*RPL9*
17	rs3833162	27,071,442	G	0.6435	0.004835	1.10E-55	cg04212500	27,184,483	*ERAL1*
20	rs3764715	1,287,051	A	− 0.3891	0.002947	1.52E-55	cg17341969	1,287,000	*SDCBP2*
1	rs6697965	220,943,801	C	0.4621	0.003507	1.67E-55	cg12466610	220,950,155	*MARCH12*
17	rs9897355	80,078,095	G	0.4414	0.003403	3.19E-55	cg25388952	80,084,596	*CCDC57*
13	rs1040961	40,108,008	G	0.4808	0.003773	6.55E-55	cg17707870	40,107,957	*AK021977*
5	rs113644940	174,915,503	T	− 0.4505	0.003674	3.16E-54	cg20462978	174,911,722	*SFXN1*
6	rs660594	31,837,250	G	0.3695	0.003024	3.66E-54	cg20370184	31,838,494	*SLC44A4*
3	rs76046201	15,365,139	T	− 0.4432	0.003671	5.98E-54	cg09627057	15,377,670	*SH3BP5*
2	rs6706223	33,944,002	G	0.4451	0.00374	1.07E-53	cg04131969	33,951,597	*MYADML*
6	rs9380143	29,802,045	T	− 0.4279	0.003698	3.38E-53	cg03570263	30,040,291	*RNF39*
6	rs138009982	170,453,220	A	0.4388	0.003921	1.33E-52	cg11400162	170,455,448	*LOC154449*
6	rs72860388	32,904,703	T	− 0.3676	0.003288	1.38E-52	cg21992044	32,918,073	*HLA-DMA*
7	rs7807520	2,087,545	C	− 0.4547	0.004083	1.63E-52	cg21598190	2,099,404	*MAD1L1*
9	rs13299342	136,141,504	A	− 0.4277	0.003848	1.74E-52	cg13683939	136,152,547	*ABO*
1	rs35195267	92,398,884	T	0.3806	0.003438	2.08E-52	cg01081438	92,417,998	*BRDT*
15	rs376992916	65,245,209	T	− 0.4207	0.003825	2.68E-52	cg25879395	65,272,510	*SPG21*
8	rs11167041	142,258,889	A	− 0.4478	0.004167	6.95E-52	cg04123498	142,283,514	*SLC45A4*
8	rs12675160	140,918,110	A	− 0.4302	0.004021	8.29E-52	cg16191297	140,926,659	*AX748239*
7	rs1108056	101,834,081	A	− 0.4235	0.003977	1.01E-51	cg18088486	101,837,098	*SH2B2*
7	rs798558	2,758,935	G	− 0.4117	0.003963	2.79E-51	cg17393140	2,764,079	*AMZ1*
1	rs12074147	40,203,722	C	− 0.4137	0.004	3.35E-51	cg07703391	40,225,995	*AB075489*
8	rs7822181	10,049,872	T	− 0.4409	0.00427	3.57E-51	cg26077133	10,049,821	*MSRA*
12	rs7139321	123,719,528	T	0.4135	0.004077	7.40E-51	cg09084244	123,757,810	*CDK2AP1*
11	rs678679	35,608,275	T	− 0.4807	0.004747	7.90E-51	cg26465155	35,611,044	*FJX1*

**Table 3:** Top 30 most significant *cis*-mQTLs in blood

CHR	SNP	BP	A1	BETA	SE	*P* value	DNAm probe Site ID	Probe start (hg19)	Closest TSS gene name
3	rs6783741	43,455,926	T	0.448	0.001743	2.21E-67	cg11035303	43,465,453	*SNRK*
6	6:29699301:TGAGAGA :TGAGA	29,699,301	TGAGAGA	0.4433	0.002662	1.16E-59	cg27230769	29,705,998	*HLA-F-AS1*
8	rs12675160	140,918,110	A	− 0.4395	0.002672	1.91E-59	cg16191297	140,926,659	*AX748239*
15	rs4776894	67,416,445	C	− 0.4491	0.002948	4.42E-58	cg07882838	67,417,557	*SMAD3*
2	rs6706223	33,944,002	G	0.4457	0.002985	1.00E-57	cg04131969	33,951,597	*MYADML*
6	rs9380143	29,802,045	T	− 0.4285	0.002918	2.00E-57	cg03570263	30,040,291	*RNF39*
13	rs1040961	40,108,008	G	0.4481	0.003182	1.10E-56	cg17707870	40,107,957	*AK021977*
4	rs5856334	16,076,153	AT	0.4034	0.002962	4.31E-56	cg17858192	16,077,757	*PROM1*
4	rs6854452	39,446,337	A	0.4776	0.003507	4.33E-56	cg19311470	39,460,490	*RPL9*
11	rs73555593	107,462,942	A	0.4321	0.003249	1.15E-55	cg22355889	107,461,585	*ELMOD1*
3	rs1054190	119,536,718	T	− 0.3963	0.002991	1.32E-55	cg12414339	119,536,718	*NR1I2*
1	rs6687657	33,599,737	T	− 0.4317	0.003277	1.68E-55	cg12386614	33,608,003	*AX747064*
7	rs62444879	2,048,470	G	− 0.4355	0.003316	1.90E-55	cg03723481	2,071,723	*MAD1L1*
11	rs10750097	116,664,040	G	0.4412	0.003384	2.59E-55	cg12556569	116,663,989	*APOA5*
19	rs1433089	52,506,985	C	− 0.435	0.003369	3.81E-55	cg01561758	52,514,395	*ZNF615*
6	rs138009982	170,453,220	A	0.4368	0.003437	7.35E-55	cg11400162	170,455,448	*LOC154449*
2	rs61702354	25,970,644	A	0.3672	0.002935	1.40E-54	cg17717333	26,101,647	*ASXL2*
14	rs35595004	52,733,244	A	− 0.4397	0.00357	2.64E-54	cg23022053	52,733,193	*PTGDR*
10	rs10900074	45,071,312	A	− 0.427	0.003469	2.70E-54	cg02113055	45,072,470	*CXCL12*
12	rs928993	52,798,364	A	− 0.4268	0.003471	2.83E-54	cg19393008	52,798,313	*KRT82*
6	rs3130978	31,082,188	A	− 0.4033	0.003301	3.65E-54	cg24926791	31,082,137	*PSORS1C1*
21	rs1721	46,349,496	T	− 0.4368	0.003577	3.76E-54	cg02464073	46,349,496	*ITGB2*
9	rs11789671	120,504,614	A	− 0.414	0.003395	3.94E-54	cg21242448	120,510,244	*TLR4*
11	rs11230502	60,607,476	A	− 0.4072	0.003374	6.09E-54	cg06394820	60,608,241	*CCDC86*
12	rs7139321	123,719,528	T	0.4466	0.00372	7.49E-54	cg09084244	123,757,810	*CDK2AP1*
7	rs1108056	101,834,081	A	− 0.4386	0.003676	9.75E-54	cg18088486	101,837,098	*SH2B2*
1	rs284307	10,739,255	C	− 0.3581	0.003024	1.33E-53	cg13387643	10,737,562	*Mir_584*
12	rs10777168	76,651,353	C	0.407	0.003444	1.43E-53	cg26864661	76,661,181	*BBS10*
20	rs6073257	42,561,422	C	0.3894	0.00333	2.19E-53	cg26365090	42,574,362	*TOX2*
16	rs12149056	58,690,964	A	− 0.4447	0.003819	2.62E-53	cg05876883	58,704,445	*SLC38A7*

### Overlap between endometrial and blood mQTLs

We were able to test how well our blood mQTL dataset reproduced previously identified mQTL signals by overlapping our signals with summary data from a meta-analysis of the Lothian Birth Cohorts (LBC) and Brisbane Systems Genetics Study (BSGS) datasets from 1980 individuals [23]. Approximately 70% of *cis*-mQTLs identified in blood in this study have been reported in blood previously. This replication shows that our blood data are consistent with larger blood mQTL datasets that themselves can act as a proxy to increase the power of subsequent analyses. Focusing on our matched endometrium and blood data, 60% of endometrial tissue *cis*-mQTLs were also found in our blood *cis*-mQTL set. Similarly, when compared to the larger LBC-BSGS blood mQTL dataset, 62% of endometrial tissue *cis*-mQTLs were also seen in a larger blood dataset. The 30 *cis*-mQTLs with the largest effect size in endometrial tissue that are also in blood are shown in Additional file 2: Figure S6, the majority displaying effect sizes in the same direction. It is important to note however that the detection of differences in effect size between tissues is dependent on sample size and the power to detect differences [24].

Using the *r*_b_ method outlined by Qi et al. [24], we estimated the correlation in genetic effects between *cis*-mQTLs in endometrium and blood, and found a high correlation between tissues from the same individuals (*r*_b_ = 0.78). This correlation was similar to the correlation in *cis*-mQTL effects between brain and blood (*r*_b_ = 0.78) in the Qi et al. [24] study.

### Overlap with reproductive traits and pathologies

#### GWAS overlap

To investigate possible endometriosis-associated disease mechanisms impacted by epigenetic regulation in the endometrium, we identified any *cis*-mQTL mSNPs (mSNP—SNP with a significant mQTL) in genomic regions previously associated with endometriosis. Five mSNPs associated with DNAm probe sites closest to *GREB1*, *C11orf46*, *NR2C1*, *KDR* and *WNT4* are located within regions associated with endometriosis risk (Table 4).

**Table 4:** Endometrial *cis*-mQTL mSNPs associated with endometriosis

CHR	SNP	BP	BETA	*P* value	DNAm probe site ID	Probe start (hg19)	Closest TSS gene name	GWAS *P* value
2	rs16857668	11,723,110	− 0.3996	2.95e-41	cg16908938	11,728,029	*GREB1*	2.345E-15
11	rs11031006	30,226,528	0.07907	2.42e-06	cg26197155	30,344,676	*C11orf46*	8.558E-08
12	rs35223035	95,675,326	0.05842	4.94e-05	cg06948737	95,471,414	*NR2C1*	*R*^2^ = 0.89 with GWAS SNP rs4762326 (*P* = 2.20E-09)
4	rs1551641	55,993,915	0.0592	0.0001091	cg07123701	56,024,384	*KDR*	3.736E-11
1	rs12405695	22,365,689	− 0.09405	0.0001206	cg03519931	22,466,137	*WNT4*	1.297E-14

Using Functional Mapping and Annotation of Genome-Wide Association (FUMA) to test for overlap between mSNPs and SNPs associated with traits and diseases in the GWAS catalogue, we identified 632 mSNPs that matched, or were in linkage disequilibrium with the SNPs in the GWAS catalogue that are significantly associated with 482 different traits and diseases (Additional file 3: Table S3). Some of the overlapping SNPs included those associated with reproductive traits and diseases such as age at first birth, birth weight, endometriosis, ovarian cancer, and age of menarche and menopause.

### Summary-data-based Mendelian randomisation

**Fig. 4:**
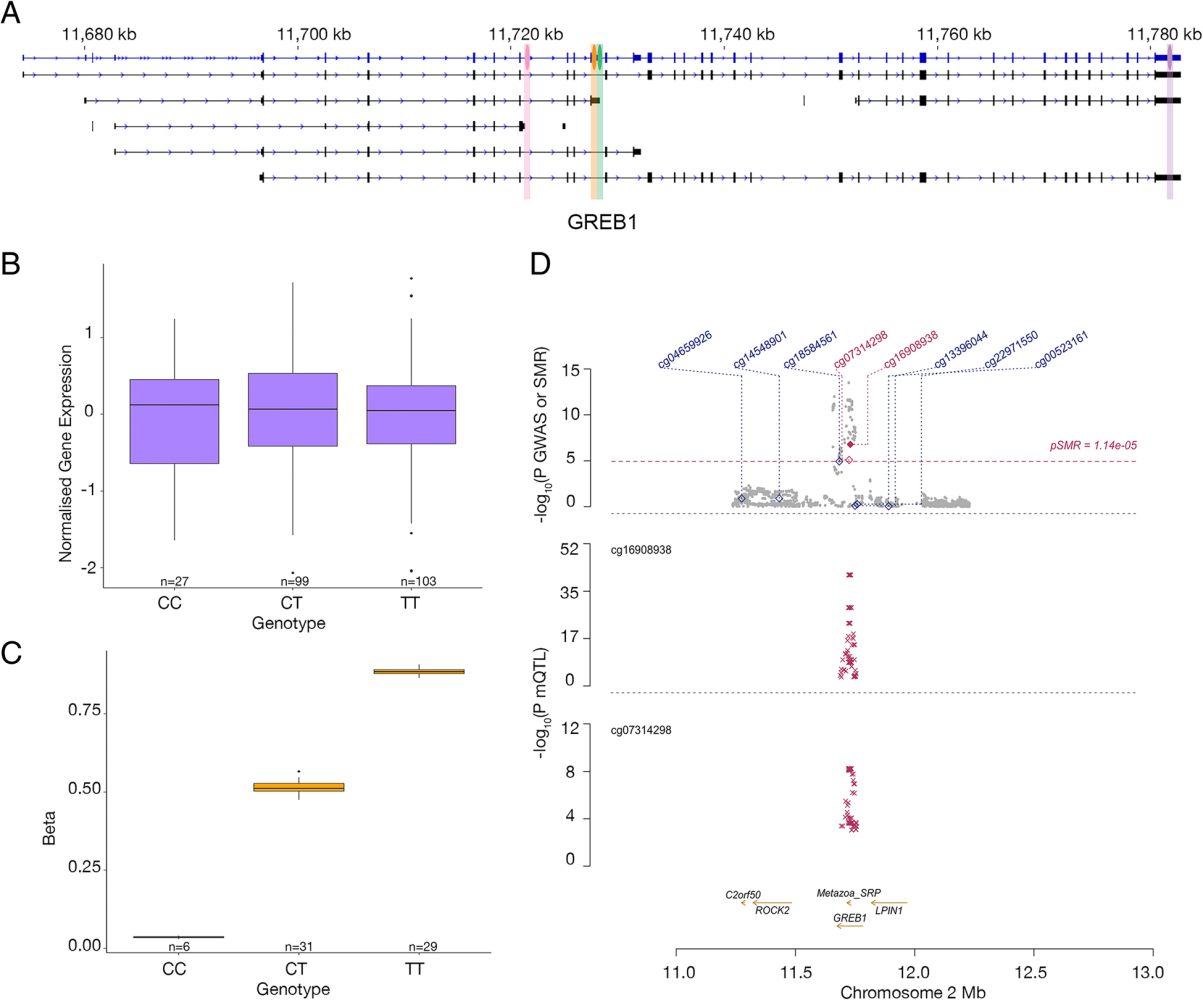
Association of methylation differences near the *GREB1* locus and endometriosis risk. **a** Location of *GREB1* transcripts on chromosome 2 with marked locations of the lead endometriosis risk SNP (rs11674184) for the *GREB1* locus (pink), the *GREB1* gene expression array probe (ILMN_1721170) position (purple), the location of mQTL DNAm probe (cg16908938) (orange) and mQTL SNP (rs59129126) (green) passing the SMR analysis. **b** Expression of ILMN_1721170 in endometrial samples from women with different genotypes at rs59129126. **c** Methylation at DNAm probe cg16908938 in endometrial samples from women with different genotypes at rs59129126. **d** SMR locus plot showing the results at *GREB1*/*Metazoa*_*SRP* locus for endometriosis. Results of the latest GWAS meta-analysis are shown in the top plot, grey dots representing the *P* values for SNPs and diamonds representing the *P* values for DNAm probe sites from the SMR test. Sites highlighted in red passed the SMR test. The middle and bottom plots show the endometrial mQTL *P* values of SNPs from this study for the DNAm probe sites nearest to *GREB1* and *Metazoa*_*SRP* respectively

To test for a causal/pleiotropic relationship between methylation status, genotype and endometriosis, we applied summary-data-based Mendelian randomisation (SMR) and heterogeneity in dependent instruments (HEIDI) methods [25] to endometriosis meta-analysis summary data from Sapkota et al. [26] and our endometrial mQTL summary data. A total of five DNAm probe sites passed the SMR test (*P*_*SMR*_ < 1.2 × 10^−5^). These five sites were annotated to growth regulating oestrogen receptor binding 1 (*GREB1*) and SNP rs59129126, *Metazoa_SRP* and SNP rs28689909, and kinase insert domain receptor (*KDR*) (3 DNAm probe sites) and SNPs rs62304733 and rs6554237 (Table 5). Only two of the five DNAm probe sites, those annotated to *GREB1* and *Metazoa*_*SRP*, were not rejected by the HEIDI test with *P*_*HEIDI*_ > 0.01 (Fig. 4).

**Table 5:** Results of the SMR analysis conducted using endometrial mQTLs and summary statistics from an endometriosis meta-analysis

CHR	DNAm probe site ID	Closest TSS gene name	Probe bp	Top SNP	Top SNP bp	A1	p_SMR	p_HET
2	cg16908938	*GREB1*	11,728,029	rs59129126	11,728,388	C	1.58E-07	1.25E-01
4	cg10360906	*KDR*	56,023,701	rs62304733	56,024,199	C	7.87E-07	9.34E-04
4	cg09978860	*KDR*	56,023,920	rs62304733	56,024,199	C	2.85E-06	8.37E-03
4	cg01777861	*KDR*	56,023,794	rs6554237	56,025,361	T	5.74E-06	8.46E-03
2	cg07314298	*Metazoa_SRP*	11,723,111	rs28689909	11,735,061	A	7.93E-06	1.26E-02

The SMR analysis was repeated using blood mQTL summary data from the larger LBC-BSGS blood dataset. Six signals passed the SMR test (*P*_*SMR*_ < 5.6 × 10^−7^) and were not rejected by the HEIDI test (*P*_*HEIDI*_ > 3.8 × 10^−3^) test. These included two DNAm probe sites closest to *GREB1*, two closest to *WNT4*, one closest to *Metazoa_SRP* and one closest to *C11orf46* (Table 6).

**Table 6:** Results of the SMR analysis conducted using blood mQTLs and summary statistics from an endometriosis meta-analysis

CHR	DNAm probe Site ID	Closest TSS gene name	Probe bp	Top SNP	Top SNP bp	A1	b_SMR	p_SMR	p_HET
2	cg02584498	*GREB1*	11,674,057	rs77294520	11,660,955	C	0.149038	1.01E-11	0.009952231
2	cg10849854	*GREB1*	11,674,557	rs77294520	11,660,955	C	0.250387	4.40E-10	0.000121199
4	cg10360906	*KDR*	56,023,751	rs11936364	56,019,253	T	− 0.075528	2.43E-09	0.000696251
4	cg01777861	*KDR*	56,023,843	rs7696256	56,023,747	G	− 0.096417	3.38E-09	0.000267448
4	cg09978860	*KDR*	56,023,921	rs11936364	56,019,253	T	− 0.0974429	3.70E-09	0.000221536
1	cg25011003	*WNT4*	22,470,341	rs55938609	22,470,451	C	0.280871	4.71E-09	0.181277
4	cg16572876	*KDR*	56,024,045	rs11936364	56,019,253	T	− 0.144697	1.17E-08	0.000408871
4	cg20092376	*KDR*	56,023,423	rs6837695	56,015,840	T	− 0.148622	1.17E-08	0.000631242
4	cg07123701	*KDR*	56,024,434	rs11936364	56,019,253	T	− 0.151501	1.29E-08	0.000349096
2	cg16908938	*GREB1*	11,728,079	rs59129126	11,728,388	C	0.0779192	1.34E-08	0.04232295
2	cg07314298	*Metazoa* *_SRP*	11,723,111	rs59129126	11,728,388	C	0.105449	1.88E-08	0.1823231
1	cg15582954	*WNT4*	22,470,343	rs55938609	22,470,451	C	0.361317	7.96E-08	0.3437667
11	cg26197155	*C11orf46*	30,344,725	rs12271187	30,319,259	A	− 0.112429	1.71E-07	0.8086896

**Fig. 5:**
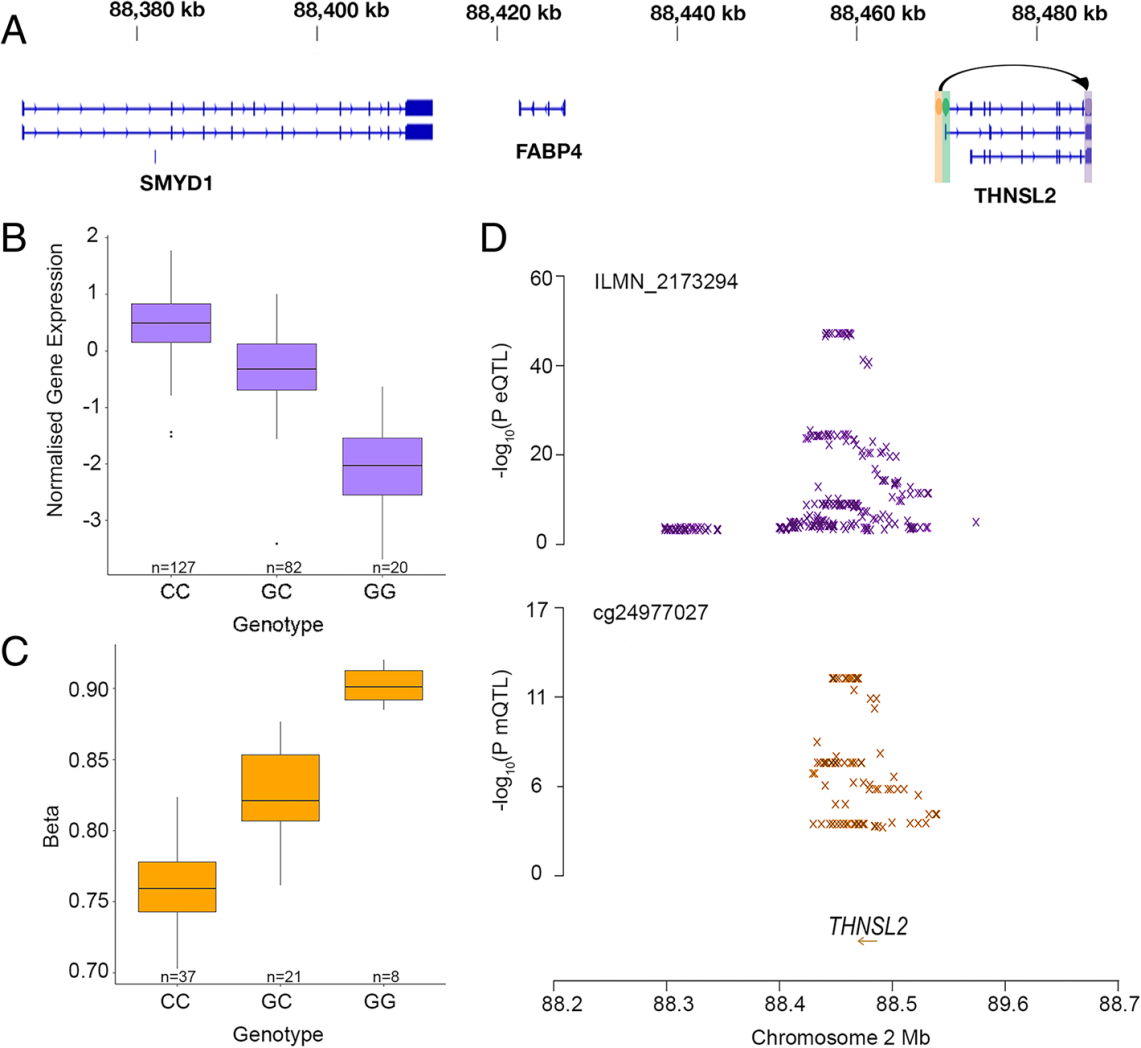
An mQTL affecting gene expression of *THNSL2* immediately adjacent to the DNAm probe. **a** Location of genes on chromosome 2 surrounding an eQTL for *THNSL2* and nearby mQTL. The location of the eQTL gene expression probe is highlighted in purple, the mQTL DNAm probe is highlighted in orange and the mQTL and eQTL SNP is highlighted in green. The arrow indicates the association of the mQTL SNP with expression of the *THNSL2* probe. **b** Expression of the *THNSL2* probe (ILMN_2173294) in endometrium from women with different genotypes at rs6547758. **c** Methylation of the cg24977027 probe in endometrium from women with different genotypes at rs6547758. **d** SMR locus plot showing the endometrial eQTL *P* values of SNPs for the *THNSL2* probe (ILMN_2173294) (top) and mQTL *P* values of SNPs from this study for the DNAm probe cg24977027 (bottom)

**Fig. 6:**
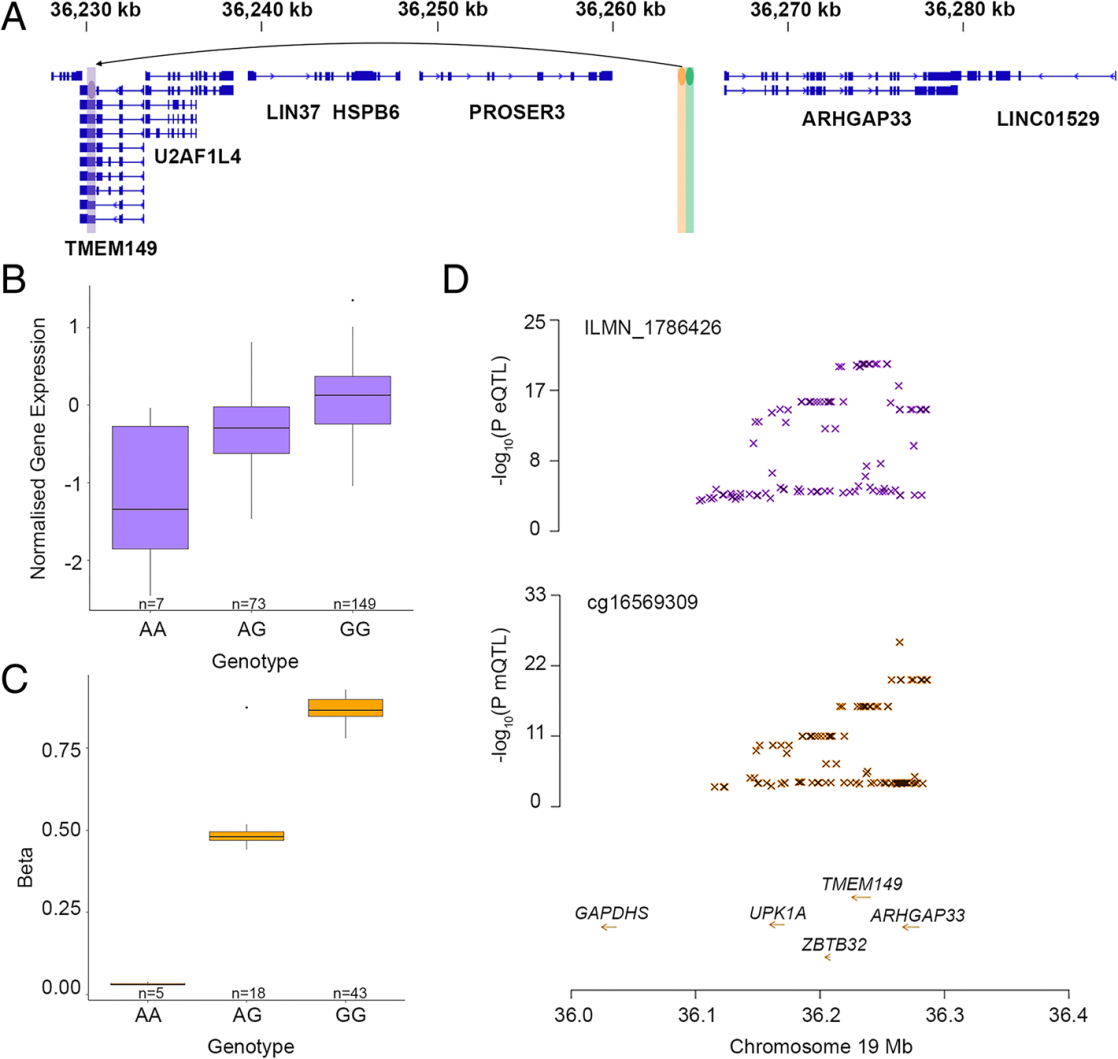
An mQTL affecting gene expression distal to DNAm probe. **a** Location of genes on chromosome 19 surrounding an mQTL closest to *ARHGAP33* and nearby eQTL for *IGFLR1*(*TMEM149*). The location of the eQTL gene expression probe is highlighted in purple, the mQTL DNAm probe is highlighted in orange and the mQTL and eQTL SNP is highlighted in green. The arrow indicates the association of the mQTL SNP with expression of the *IGFLR1* probe. **b** Expression of the *IGFLR1* probe (ILMN_1786426) in endometrium from women with different genotypes at rs62112162. **c** Methylation of the cg16569309 probe in endometrium from women with different genotypes at rs62112162. **d** SMR locus plot showing the endometrial eQTL *P* values of SNPs for the *IGFLR1* probe (ILMN_1786426) (top) and mQTL *P* values of SNPs from this study for the DNAm probe cg16569309 (bottom)

Using a multi-omic approach within the SMR software and endometrial eQTL data from Fung et al. [27]; we integrated both our endometrial mQTL dataset and the eQTL dataset to identify any association between genetic regulation of a methylation site and transcription of a gene and vice versa. We used endometrial expression quantitative trait loci (eQTLs) as the outcome and endometrial mQTLs as the exposure (M2T analysis [8]) and identified 472 associations between 414 methylation probes and 186 gene expression probes (Additional file 3: Table S4). Alternatively using endometrial mQTLs as the outcome and endometrial eQTLs as the exposure (T2M analysis), we identified 353 associations between 308 methylation probes and 157 gene expression probes that passed the SMR and HEIDI tests (Additional file 3: Table S5). We observed 275 associations overlapping between M2T and T2M analyses, the majority of loci showing opposite directions of effect (Additional file 1: Supplementary Note 2). This is consistent with both pleiotropy and the hypothesis that genotypes can regulate gene expression by altering the methylation at nearby DNAm probe sites and also can potentially affect methylation at DNAm probe sites via changes in gene expression. An estimated ~ 26% of the DNAm probe sites targeted the closest gene whilst the remaining sites target more distant genes. An example of a DNAm probe site targeting the most immediate gene, threonine synthase like 2 (*THNSL2*), is shown in Fig. 5, both the DNAm probe site and associated SNP located within the *THNSL2* promotor. An example of a DNAm probe site targeting a more distant gene, IGF-like family receptor 1 (*IGFLR1*/*TMEM149*), is shown in Fig. 6. We mapped the position of the associated M2T DNAm probe sites, which also have mQTLs in blood, to annotated regulatory regions and found that ~ 90% were within known regulatory elements. M2T DNAm probes were significantly enriched in promoters (fold-change = 1.52, *P* = 2.18 × 10^−8^) and were significantly less represented in quiescent regions (fold-change = 0.57, *P* = 9.78 × 10^−9^) when compared to randomly sampled probes with matched variance (Additional file 2: Figure S7).

SMR was also used to test for any associations between endometrial eQTLs and various other traits and diseases. We found pleiotropic associations between 409 probes and 17 traits including those relating to reproductive biology, age at menopause and ovarian cancer (Additional file 3: Table S6). Approximately 63% of mQTLs that passed the SMR test and were not rejected by the HEIDI test for these traits were also present in blood. However, for mQTLs associated with menopause and ovarian cancer, only 6 of the 26 mQTLs were also in blood. This suggests that tissue-specific effects may contribute to these phenotypes.

### Functional annotation

Gene pathways potentially impacted by changes in methylation in endometrium were investigated using the pathway enrichment analysis in FUMA. No MsigDB Hallmark pathways were enriched for genes with transcription start sites closest to DNAm probe sites differentially methylated between stages of the menstrual cycle. Significantly enriched pathways for overlapping gene sets between differentially methylated and differentially expressed genes include epithelial mesenchymal transition, oestrogen response, *IL2 STAT5* signalling and *TNFA* signalling via *NFKB* (Additional file 2: Figure S8).

To identify gene pathways potentially affected by genetic regulation of methylation in endometrium and/or blood, we also conducted a pathway analysis of genes annotated to *cis*-mQTL probes. Pathway analysis showed that ultraviolet (UV) response, early oestrogen response and epithelial mesenchymal transition were the most significantly enriched hallmark pathways in both endometrial tissue and blood; GO biological processes such as intracellular signal transduction, regulation of cell differentiation and positive regulation of molecular function were also highly enriched in both tissues (Table 7). The majority of enriched hallmark pathways were consistent across both blood and endometrium with the exception of pancreas beta cells, hedgehog signalling and the *PI3K*/*AKT*/*MTOR* signally pathways, which were only enriched in blood mQTLs, and peroxisome and angiogenesis pathways that were only enriched in endometrium (Additional file 3: Table S7 and S8). Similarly, whilst 85% of the enriched GO biological process pathways are shared between blood and endometrium, there are some more biologically relevant pathways that are tissue specific such as artery development, lymphocyte differentiation and cardiac cell development in blood and regulation of meiotic cell cycle, regulation of epithelial structure maintenance and regulation of embryonic development in endometrium (Additional file 3: Table S7 and S8).

**Table 7:** Top hallmark and GO biological processes pathways enriched for genes closest to DNAm probe sites in *cis*-mQTLs in endometrium and blood

Tissue	GeneSet	*N*	*n*	*P* value	Adjusted *P*
Endometrial mQTLs	Go intracellular signal transduction	1568	237	1.92E-29	5.50E-26
	Go regulation of multicellular organismal development	1667	247	2.48E-29	5.50E-26
	Go regulation of transport	1799	257	5.10E-28	7.54E-25
	Go regulation of cell differentiation	1488	224	1.29E-27	1.43E-24
	Go regulation of cell proliferation	1492	221	2.73E-26	2.42E-23
	Go neurogenesis	1401	211	4.37E-26	3.23E-23
	Go regulation of cell death	1471	218	5.66E-26	3.58E-23
	Go locomotion	1111	179	1.16E-25	6.41E-23
	Go positive regulation of molecular function	1786	248	3.00E-25	1.48E-22
	Go regulation of transcription from rna polymerase ii promoter	1780	246	9.31E-25	4.13E-22
	Hallmark uv response dn	144	37	9.77E-13	4.88E-11
	Hallmark oestrogen response early	200	41	1.94E-10	4.84E-09
	Hallmark epithelial mesenchymal transition	199	34	6.07E-07	8.57E-06
	Hallmark complement	200	34	6.86E-07	8.57E-06
	Hallmark androgen response	100	21	1.68E-06	9.69E-06
	Hallmark hypoxia	200	33	1.94E-06	9.69E-06
	Hallmark allograft rejection	200	33	1.94E-06	9.69E-06
	Hallmark il2 stat5 signalling	200	33	1.94E-06	9.69E-06
	Hallmark p53 pathway	200	33	1.94E-06	9.69E-06
	Hallmark myogenesis	200	33	1.94E-06	9.69E-06
Blood mQTLs	Go intracellular signal transduction	1568	330	9.02e-46	4.00e-42
	Go positive regulation of molecular function	1786	345	2.00e-39	4.44e-36
	Go neurogenesis	1401	291	3.12e-39	4.62e-36
	Go regulation of multicellular organismal development	1667	326	2.39e-38	2.29e-35
	Go regulation of hydrolase activity	1325	278	2.58e-38	2.29e-35
	Go regulation of intracellular signal transduction	1651	322	1.23e-37	9.08e-35
	Go positive regulation of catalytic activity	1515	302	3.82e-37	2.12e-34
	Go tissue development	1508	301	3.91e-37	2.12e-34
	Go locomotion	1111	244	4.78e-37	2.12e-34
	Go regulation of cell differentiation	1488	298	4.79e-37	2.12e-34
	Hallmark uv response dn	144	48	5.31e-16	2.66e-14
	Hallmark oestrogen response early	200	56	1.74e-14	4.35e-13
	Hallmark epithelial mesenchymal transition	199	48	3.49e-10	5.25e-09
	Hallmark complement	200	48	4.20e-10	5.25e-09
	Hallmark oestrogen response late	200	46	4.04e-09	4.04e-08
	Hallmark myogenesis	200	45	1.20e-08	1.00e-07
	Hallmark mitotic spindle	200	44	3.48e-08	2.49e-07
	Hallmark p53 pathway	200	43	9.80e-08	6.12e-07
	Hallmark apical junction	199	42	2.32e-07	1.22e-06
	Hallmark tnfa signalling via nfkb	200	42	2.68e-07	1.22e-06

Both blood and endometrium *cis-*mQTLs had very similar methylome patterns in the context of CpG locations; an average of 47% of *cis*-mQTLs were located in open sea regions of the genome followed by 24% located in shores and 17% in CpG islands (Additional file 3: Table S9, Additional file 2: Figure S9). mQTL DNAm probes were also annotated to predicted regulatory regions, the majority located in promoters and quiescent regions (Additional file 1: Supplementary Note 2).

Using available data on the genomic location of oestrogen receptor (ESR) binding sites, we identified 414 differentially methylated DNAm probes that overlapped *ESR* binding sites (Additional file 3: Table S10). We also identified 791 *cis*-mQTL mSNPs that were within *ESR* binding sites (Additional file 3: Table S11). Pathway analysis identified that the early (*P* = 5.16 × 10^−12^) and late (*P* = 5.43 × 10^−5^) oestrogen response pathway and the cholesterol homeostasis pathway (*P* = 9.18 × 10^−6^) were most significantly enriched for genes closest to these mQTL DNAm probe sites.

## Discussion

We analysed genetic control of methylation in human endometrium and compared results with methylation in DNA from blood samples collected from the women at the same time. We observed marked changes in DNAm in the endometrium across the menstrual cycle for some probes in agreement with previous studies [19–21]. The 66 women were sampled at different stages of the menstrual cycle and 6% of DNAm probe sites (27,262 sites) showed evidence of differential methylation across the cycle. The endometrium is a biologically and transcriptionally dynamic tissue with significant changes in gene expression across the menstrual cycle [15, 17, 28]. Genes previously reported as differentially expressed across the cycle that were also assigned to differentially methylated sites across the menstrual cycle were significantly enriched in the oestrogen response pathway and 1.5% of differentially methylated probes are located in *ESR* binding sites. Oestrogen plays a major role in regulating proliferation of epithelial and stromal cells during the proliferative phase of the menstrual cycle [29–31]. Differences in methylation between cycle stages were not observed in matched blood samples from the same women. Therefore, our findings highlight tissue-specific features of methylation signals in endometrium, although it is not known if the differential methylation is a reflection of differential methylation between cell types and the changes in the cellular composition across the cycle.

We identified 4546 sentinel *cis*-mQTLs and 434 sentinel *trans*-mQTLs in endometrial tissue samples. There was a high correlation of genetic effects (*r*_b_ = 0.78) and overlap (~ 60%) in mQTLs between endometrium and blood samples from the same women and results were similar when comparing with a much larger sample of mQTLs in blood samples from unrelated individuals [23]. Of interest were the subset of mQTLs not present in blood and that overlapped oestrogen receptor binding sites, suggesting possible tissue-specific effects. Two examples were mQTLs at loci near the G protein-coupled oestrogen receptor 1 (*GPER*) and Plectin (*PLEC*)*. GPER* is a membrane protein from the seven-transmembrane (7TM) GPCR family, localised to the endoplasmic reticulum [32]. This receptor mediates both rapid non-genomic signalling cascades and transcriptional changes that regulate cell proliferation and apoptosis in response to oestrogen [32, 33]. *GPER* has the potential to play an important regulatory role in the proliferation and regeneration of endometrium in response to an increase in circulating oestrogen during the proliferative phase of the menstrual cycle. *PLEC* belongs to a family of proteins that function as cytolinkers/plakins and play an important role in maintaining cytoskeleton structure and subsequently cell and tissue integrity, and cell adhesion [34]. *PLEC* is upregulated from the early to mid-secretory stage of the cycle in normal women and decreases again from mid to late-secretory stage during end of receptive period [35]. *PLEC* has been reported as downregulated in endometrium of women with repeated embryo implantation failure [36], and in women with endometriosis during the window of implantation [37], suggesting an important role in female fertility. The differences in DNA methylation across the menstrual cycle and mQTLs specific to the endometrium support the need for both tissue-specific studies and comparisons between tissues to understand regulation of epigenetic signals and their role in disease. However, much larger studies in target tissues such as endometrium will be necessary to have sufficient power to detect the tissue-specific mQTLs that may be associated with genetic effects on disease risk.

SMR analysis identified significant overlap of mQTLs with five endometriosis GWAS signals. Results include new evidence that the risk SNPs on chromosome 2 alter methylation at DNAm probe sites located within 350 bp of the *GREB1* transcription start site in blood and another within the 3′UTR of a *GREB1* transcript in both endometrium and blood. *GREB1* is an oestrogen-responsive gene involved in the oestrogen receptor-regulated pathway, essential for oestrogen receptor transcription [38]. GREB1 has also been reported to regulate proliferation in breast, prostate, and ovarian cancers [39–42].

Evidence for mQTLs near *GREB1* is an interesting result as we did not detect any genetic effects on *GREB1* gene or protein expression in endometrium previously [17, 43]. Changes in methylation can result in alternative splicing [44] and the risk SNPs may alter methylation and expression of particular *GREB1* transcripts that could not be individually identified from the microarray. The absence of an eQTL for *GREB1* could also suggest epigenetic regulation of post-transcriptional modifications through mechanisms including microRNA regulation of *GREB1* [45, 46], association with protein QTLs (pQTLs) independent of mRNA expression [47, 48], RNA folding, accessibility of functional sites [49, 50] or post-transcriptional modifications such as N^6^-methyladenosine (m^6^A) methylation which are enriched in stop codons and 3′UTRs [45, 51, 52]. Investigation into transcript-specific and post-transcriptional effects at this locus are needed to confirm any effects on *GREB1*. The absence of one of the SMR significant mQTLs (cg02584498- rs77294520) in endometrium may indicate tissue-specific effects or limited power in our endometrial dataset to detect the mQTL.

Three probes nearest to *KDR* also passed the SMR analysis in both endometrium and blood, but were rejected by the HEIDI test, suggesting significant heterogeneity and the possibility of multiple causal variants. *KDR* is a vascular endothelial growth factor receptor involved in the proliferation and differentiation of endothelial cells with a potential role in implantation and successful pregnancy [53–55]. The association of *GREB1* and *KDR* mQTLs with endometriosis risk in both endometrium and blood may suggest the biological mechanisms that increase endometriosis risk may not be specific to endometrium.

The SMR analyses also detected target genes for DNAm probe sites with pleiotropic associations for mQTLs associated with age at menopause and ovarian cancer, many of which were not replicated in blood. These associations may implicate tissue-specific mQTLs, such as those in oestrogen-responsive tissues, in reproductive disease. Some examples of instances whereby genomic regulation of methylation may influence reproductive traits and pathologies include the *ZNF346/UIMC1*, *SYCP2L* and *HOX* gene loci. The *ZNF346*/*UIMC1* locus is strongly associated with age at menopause and forms part the *BRCA1*-*A* complex, which regulates oestrogen receptor transcription and DNA repair, both of which are important in regulating endometrial oestrogen response and meiosis [56, 57]. The *SYCP2L* locus associated with age at menopause also had a pleiotropic relationship with an endometrial mQTL in this region. *SYCP2L* is a paralog of the synaptonemal complex protein 2 and is known to localise to centromeres in oocytes and promote primordial oocyte survival [57, 58]. Finally, a locus surrounded by *HOX* genes on chromosome 2 and containing an endometrial mQTL has been associated with epithelial ovarian cancer; many of the *HOX* genes in the region are known to regulate embryogenesis and neoplastic development [59]. The significant associations between endometrial mQTLs and various traits and diseases highlight the importance of our findings in the broader scientific community, identifying genetic regulatory mechanisms that are contributing to disease phenotypes.

We also identified pleiotropic associations between methylation at 414 methylation probes and altered expression for 186 gene expression probes, where both are associated with a shared causal variant. DNAm probes associated with gene expression were enriched in promoters, supporting the hypothesis that DNAm probe sites located in regulatory regions can affect gene expression of the associated genes in endometrium, as shown previously in blood [8]. The high proportion of DNAm probe sites (> 70%) mapping to distant genes is important when interpreting the mechanisms behind transcription regulation. DNAm probe sites located further away from their target gene may reside in regulatory regions such as enhancers that can interact with distant target genes through mechanisms such as chromatin looping [60, 61]. One example of a distant target, *IGFLR1*(*TMEM149*), has expression associated with a SNP and DNAm site ~ 35 kb downstream. The locus containing the SNP and DNAm is located within 400 bp of a regulatory region, a CTCF binding site, annotated in the Ensembl regulatory build [62]. CTCF protein is known to mediate chromatin looping and may represent a possible mechanism of regulation of *IGFLR1* expression [63, 64]. Most disease-associated loci are within non-coding regions [65–67], including loci associated with the endometrial-related diseases endometriosis [26] and endometrial cancer [68]. Therefore, understanding how regulatory regions in these non-coding regions can affect distant target genes is important in understanding both endometrial biology and disease mechanisms.

An important limitation of this study is the small sample size which limits the statistical power to detect robust differences in methylation in endometrium. Previous epigenetic studies using the 450 k methylation beadchip and a significance level of *P* = 2.4 × 10^−7^ reported that 59 and 186 samples would have 80% power to detect mean differences in methylation of 15% and 8% respectively [69]. Power calculations by Rahmioglu et al. [21] show that 500 samples are needed to detect mean changes in methylation of 2% with 80% power in endometrium. Using the pwr.t2n.test function in R, we calculated that we had 80% power to detect a mean difference in methylation of 10% at a significance level of *P* = 0.05 and 25% at a genome-wide significance level of *P* = 1.13 × 10^−7^. Using variance estimates from our data, we estimated that 155 samples could detect a mean difference of 8% and ~ 3000 samples would be required to detect a mean methylation differences of 2%, similar to estimates by Saffari et. el [69] (Additional file 3: Table S12). Larger sample sizes would be needed to detect smaller effects of disease and menstrual cycle phase on methylation in endometrium. However, even with a limited sample size, we were able to detect over 4000 significant endometrial *cis*-mQTLs, the majority of which were previously reported in larger blood studies [23].

Another limitation of this study is results have not been adjusted for change in cell composition. There is currently no published method of estimating cell type composition in endometrial samples. Differences in methylation between menstrual cycle phases reported in this study are also likely to reflect changes in cell composition. Due to the complexity of endometrial tissue and the presence of several different cell types including stromal cells, epithelial cells and infiltrating immune cells, development of an accurate cell composition correction requires extensive research to characterise omic signatures of the individual cell types and validate a method to correct for differences in cell composition in endometrium.

## Conclusions

This is the first study to identify mQTLs in human endometrium, and shows significant overlap and correlation between mQTLs seen in endometrium with those observed in blood from the same and independent individuals. The high degree of overlap supports the use of large blood mQTL datasets as a proxy for endometrium to increase power to detect target genes for endometrial traits and diseases. There was evidence for variation in genome-wide methylation profiles across the menstrual cycle for a proportion of sites in human endometrium, changes not observed in blood, and our results highlight possible tissue-specific effects for mQTLs and enriched pathways not shared between blood and endometrium. We show that genomic regulation of methylation in endometrium has the potential to influence endometrial biology and overlap of mQTLs with risk loci for endometriosis and ovarian cancer indicate a role of methylation in reproductive diseases. Larger sample sizes are needed to identify effects of disease on methylation in endometrium and identify tissue-specific mQTLs that may be involved in endometrial biology and disease.

## Methods

### Sample collection

A total of 66 women of reproductive age (31.08 ± 6.64 years) and from European ancestry were selected for inclusion in the study. Women were recruited when attending clinics at the Royal Women’s Hospital in Melbourne, Australia, following informed written consent. The study was approved by the Human Research Ethics Committees of the Royal Women’s Hospital, Melbourne, the QIMR Berghofer Medical Research Institute and The University of Queensland (Projects 11-24 and 16-43). All sample and data collection was performed in accordance with institutional approved guidelines and regulations.

The clinical history for each participant was obtained alongside surgical notes and pathology results. Whole blood samples were collected prior to surgery. Endometrial tissue was collected by curettage during laparoscopic surgery for investigation of recurring pelvic pain and/or infertility. Forty-five of the 66 women were diagnosed with endometriosis. All women were free from exogenous hormone treatment in the 3 months prior to surgery. Menstrual cycle stage for each participant was categorised by an experienced pathologist into menstrual (M) = 3, early proliferative (EP) = 1, mid proliferative (MP) = 27, late proliferative (LP) = 5, early secretory (ES) = 6, mid secretory (MS) = 14 and late secretory (LS) = 10.

### DNA extraction, methylation array and genotyping

Buffy coat was isolated from whole blood for DNA extraction using a salting out method [70]. Endometrial tissue samples were stored in RNA*later* (Life Technologies, Grand Island, NY, USA) at − 80 °C until RNA/DNA extraction. Genomic DNA was extracted from homogenised endometrial tissues using the AllPrep DNA/RNA mini kit according to the manufacturer’s instructions (QIAGEN, Valencia, CA).

Bisulfite conversions were performed in 96-well plates using the EZ-96 DNA Methylation Direct Kit (Zymo Research, Irvine, CA, USA). Prior to conversion, DNA concentrations were determined by Quant-iT™ PicoGreen™ dsDNA Reagent (Life Technologies, Carlsbad, CA, USA) and standardised to 500 ng DNA per sample. Bisulfite converted DNA samples were hybridised to Illumina Human Methylation 450 BeadChips using the Infinium HD Methylation protocol and Tecan robotics (Illumina, San Diego, CA, USA). Samples were scanned using an Illumina iScan Reader. Methylation at each site was measured as a ratio of the intensities of methylated and un-methylated alleles at the DNAm probe site represented as β values [71].

Genomic DNA extracted from whole blood was genotyped on the HumanCoreExome chips and Infinium PsychArray (Illumina Inc., San Diego) [27]. Genotype data was filtered using the program PLINK ver 1.9 [72, 73]. SNPs not genotyped in at least 95% of individuals were removed (-geno 0.05 command) along with SNPs with a minor allele frequency (MAF) < 0.05 (-maf 0.05 command) and with Hardy-Weinberg Equilibrium (HWE) *P* < 1 × 10^−6^ (-hwe 0.000001 command). A total of 282,625 SNPs were used for imputation using the 1000 Genomes Phase 3 V5 reference panel. Genotypes were phased with ShapeIt V2 prior to imputation on the Michigan Imputation Server [74]. Additional quality control was performed on imputed genotypes to remove SNPs of poor quality (*R*^2^ < 0.8) or low MAF < 0.05, leaving 5,162,603 autosomal SNPs for subsequent analysis.

### Methylation quality control and normalisation

Quality control and normalisation of raw methylation data was performed separately for blood and tissue samples using the R package “Meffil” [75]. Genotype data present in the methylation array data was compared to genotypes of the same samples run on the HumanCoreExome chips and Infinium PsychArray (Illumina Inc., San Diego). Genotypes for all samples matched 65 corresponding SNP probes on the microarray confirming no sample error between the methylation profiles for endometrium and blood. QC parameters outlined in the Meffil manual were used for the blood and endometrial dataset (https://github.com/perishky/meffil). DNAm probes that did not exceed the background signal and met a detection *P* value of < 0.01 in > 10% of samples were removed (220 DNAm probes in endometrial tissue and 184 DNAm probes in blood) alongside probes with low bead numbers in > 10% of samples (418 DNAm probes in endometrial tissue and 346 DNAm probes in blood). There were no sample outliers with poor probe detection with > 90% of DNAm probes detected in all samples. Using control probes, ten principal components were used to adjust the methylation levels for technical effects. DNAm probe sites found to target multiple genomic regions as previously annotated by Price et al. [76] were also removed. Functional normalisation was applied to remove global differences in methylation data and to extend quantile normalisation of control probes across the data.

### DNAm principle component analysis and covariate effects

The presence of potentially cofounding sources of variation in the data was investigated through principal component (PC) analysis of DNAm profiles and association of top PCs with known covariates for both blood and tissue datasets. Principal components were computed from normalised methylation profiles for endometrium and blood. A significant association between methylation beadchip (*P* = 2.14 × 10^−6^) and PC two and between stage of cycle (*P* = 3.19 × 10^−4^) and PC one was observed in endometrium. We also detected a significant association between methylation beadchip (*P* = 1.92 × 10^−15^) and age (*P* = 0.008), with PC two and three respectively, in blood. No significant effect of endometriosis status was observed. All covariates were corrected for in later analyses where appropriate.

### Differential DNA methylation

To identify changes in the methylation state of DNAm probe sites between stages of the menstrual cycle, we performed a differential methylation analysis on both the blood and endometrial tissue methylation datasets. To increase the number of samples within each group, and power for subsequent analyses, we combined menstrual cycle stages into three main phases; menstrual (M) stage (*n* = 3), EP, MP and LP stages were merged into the proliferative (P) phase (*n* = 33) and the ES, MS and LS stages were merged into the secretory (S) phase (*n* = 30). We subsequently removed M stage samples from the differential analysis due to the small sample size and limited power. Following QC of the methylation data, 443,101 DNAm probe sites for blood and 443,016 DNAm probe sites for endometrial tissue were retained for inclusion in the cycle stage analysis. We used the eBayes method implemented in the limma package to compute a moderated *t* statistic and fold change between P and S phases.

To test for any confounding effects of endometriosis status, we also tested for differently methylated DNAm probes between women with and without endometriosis. Tests were conducted using the eBayes method for individuals in the same menstrual cycle phase and using all samples by including stage of cycle as an additional covariate. No differentially methylated DNAm probes were detected.

### mQTL analysis

We tested the association between genotype and DNAm probe site methylation in both blood and endometrial tissue datasets to identify mQTLs. All 443,101 DNAm probe sites in blood and 443,016 DNAm probe sites in endometrium passing QC were included in the mQTL analysis. Associations between 5,162,603 SNP genotypes and normalised methylation intensities were tested using a linear regression model in the software PLINK ver 1.9 (-linear command). Covariates, including age, presence of endometriosis, stage of cycle and methylation chip, were adjusted for in the analysis. The distance distribution of significant (FDR < 0.05) mQTL SNPs from their associated DNAm probes within 1 Mb showed that the vast majority of SNPs (92%) were within 250 kb of the probe site (Additional file 2: Figure S10). To capture the majority of *cis*-mQTLs and limit multiple testing, *cis*-mQTLs were defined as ± 250 kb between the SNP and the DNAm probe site start position. *Trans*-mQTLs were defined as associations between a SNP and DNAm probe site located on different chromosomes. To identify secondary independent *cis*-mQTL signals, we performed conditional analysis on sentinel *cis*-mQTLs that met a Bonferroni significance threshold of *P* < 1.13 × 10^−10^. The conditional analysis was conducted by repeating the association analysis between genotype and DNAm probe site methylation conditioning on the primary SNP.

### Context-specific mQTL analysis and overlap with differentially methylated DNAm probe sites

We tested for overlap between the differentially methylated DNAm probe sites and mQTLs. To test for interaction between genotype and stage of cycle on DNAm probe site methylation, we used the context-specific analysis method outlined by Fung et al. [17]. Briefly, we used linear regression to test for interaction between stage and genotype using the observed normalised methylation level of a probe as the dependent variable and fitting the regression coefficient of the genotype, regression coefficient of the stage of cycle and the regression coefficient of the interaction between genotype and stage of cycle. We tested 23 *cis*-mQTL probes passing Bonferroni correction in endometrial tissue that corresponded to genes differentially methylated between P and S phases of the menstrual cycle.

### Overlap between endometrial and blood mQTLs

Using blood mQTLs detected in a large meta-analysis of the Lothian Birth Cohorts (LBC) and Brisbane Systems Genetics Study (BSGS) datasets consisting of 1980 individuals [23], we were able to assess the overlap between our endometrial and blood mQTLs and those from a more highly powered study in blood. The LBC-BSGS dataset consists of 94,338 sentinel *cis*-mQTLs with a significance of at least *P* < 5 × 10^−8^ and SNPs within 2 Mb distance from each probe. mQTLs were considered to overlap if they had the same probe and associated SNP. Additionally, overlap was defined in terms of linkage disequilibrium (LD) *r*^2^ > 0.7 between the mSNP in the LBC-BSGS dataset and the endometrial mSNP based on the 1000 Genome phase 3 reference panel.

Tissue specificity of endometrial mQTLs was investigated by identifying the presence of mQTLs found in endometrial tissue in blood. Tissue mQTLs were tested for overlap with both the blood mQTL set from this study and the larger LBC-BSGS dataset. Overlap was defined in the same manner as described previously.

The correlation of *cis*-mQTL effects between endometrium and blood from the same individuals was estimated using the *r*_b_ method developed by Qi et al. [24]. mQTL effect sizes and standard errors were standardised between tissues based on z-statistics using the method described in Zhu et al. [25]. Top *cis*-mQTLs (*P* < 5 × 10^−8^) were taken from the LBC-BSGS blood dataset as an independent reference set. The top *cis*-mQTLs from the reference set were then extracted from our endometrium and blood set; *cis*-mQTLs not present in our sets were excluded and the remaining *cis*-mQTLs were used to estimate the correlation.

### Overlap with endometriosis risk loci and reproductive traits

#### GWAS overlap

Summary data available from Sapkota et al. [26] generated from ~ 15,000 European endometriosis cases were used to test overlap with our endometrial mQTLs. Overlap was determined if sentinel mQTL mSNPs matched those identified at the 19 endometriosis risk loci or if sentinel mQTL mSNPs had a minimum LD of *r*^2^ > 0.7 with the GWAS SNP. The Functional Mapping and Annotation of Genome-Wide Association (FUMA) SNP2GENE function was also used to test mSNPs for association with other traits and diseases from the GWAS catalogue.

#### Summary-data-based Mendelian randomisation

Using SMR software developed by Zhu et al. [25], we tested for pleiotropic association between DNAm probe site methylation and endometriosis. Summary data from the Sapkota et al. [26] meta-analysis was used alongside summary data from mQTLs identified in this study as input for the analysis. A total of 4546 DNAm probe sites reaching Bonferroni significance were included in the analysis and a study-wide significance threshold of *P*_SMR_ = 1.10 × 10^−5^ was applied. Heterogeneity of SMR estimates at surrounding SNPs (in LD with the top cis-mQTL) was tested using HEIDI which is incorporated in the SMR software. A *P*_HEIDI_ of < 0.05/m, where *m* is the number of probes passing the SMR test, was used to suggest heterogeneity of SMR estimates in the *cis*-region. The SMR analysis was repeated in blood using summary data from the large blood LBC-BSGS mQTL dataset [23].

The SMR software also allows the integration of multiple-omic datasets to infer a likely regulatory mechanism. We used this multi-omic analysis option to test the association between endometrial mQTLs from this study and eQTLs from our previous study [27], using only probes that passed Benjamini-Hochberg false discovery rate (FDR) cut off of 0.05. This analysis was performed using gene expression as the outcome and methylation as the exposure in a M2T analysis and was performed again using expression as the exposure and methylation as the outcome in a T2M analysis, as previously described in Wu et al. [8].

To investigate the possible impact of endometrial mQTLs in other phenotypes, we conducted further SMR analyses using GWAS summary datasets for a range of traits including body mass index (BMI), body fat percentage, leptin, lipid levels including high-density lipoprotein (HDL), low-density lipoprotein (LDL), total cholesterol (TC) and triglycerides (TG), coronary artery disease, heart rate, rheumatoid arthritis, celiac disease, inflammatory bowel disease, ulcerative colitis, type 1 diabetes, type 2 diabetes, glucose levels, insulin levels, attention deficit hyperactivity disorder (ADHD), Alzheimer’s, schizophrenia, bipolar disorder, major depressive disorder, autism, motor neurone disease, age-related macular degeneration and osteoporosis. We also included reproductive traits such as maternal birth weight, age of menopause, maternal gestational weight gain and epithelial ovarian cancer [57].

### Functional annotation

Applying locational data for *ESR* binding sites previously identified by Carrol et al. [77], we sought to identify any overlaps between differentially methylated DNAm probes and sentinel mSNPs for *cis*-mQTLs and *ES*R binding sites. Regulatory elements within which mQTL loci may act were annotated using data available from the Roadmap Epigenomics Mapping Consortium (REMC) and ENCODE [78, 79]. Due to the absence of chromatin state information for endometrium, we used chromatin state model based imputation data for 23 blood cell lines from 127 epigenomes in which 12 histone-modification marks were used to predict 25 chromatin states [80]. Functionally similar annotations were combined into 14 categories as suggested by Wu et al. [8]. Endometrial mQTLs also identified in blood were annotated to the 14 categories of functionally similar chromatin states. Using the method outlined in Wu et al. [8], we performed an enrichment analysis to test for enrichment of DNAm probes significantly associated with gene expression in the M2T analysis, in the 14 functional categories.

### Pathway analysis

To identify pathways potentially affected by or regulating changes in methylation across the cycle, we performed a pathway analysis using the “GENE2FUNC” function on the FUMA GWAS web-based platform [81]. DNAm probe sites differentially methylated between phases of the menstrual cycle were annotated to the nearest TSS, and the resulting gene lists were used as input for the pathway analysis. The same was done for *cis*-mQTLs meeting Bonferroni genome-wide significance.

## Additional files

Additional file 1: Supplementary Notes. (PDF 65 kb)

Additional file 2: Supplementary Figures. (PDF 4540 kb)

Additional file 3: Supplementary Tables. (XLSX 4830 kb)

## Abbreviations

ADHD: Attention deficit hyperactivity disorder
BMI: Body mass index
BSGS: Brisbane Systems Genetics Study
DNAm: DNA methylation
EP: Early proliferative
eQTL: Expression quantitative trait loci
ES: Early secretory
ESR: Oestrogen receptor
FDR: False discovery rate
HDL: High-density lipoprotein
HEIDI: Heterogeneity in dependent instruments
HWE: Hardy-Weinberg equilibrium
LBC: Lothian Birth Cohorts
LD: Linkage disequilibrium
LDL: Low-density lipoprotein
LP: Late proliferative
LS: Late secretory
M: Menstrual
MAF: Minor allele frequency
MP: Mid proliferative
mQTL: Methylation quantitative trait loci
MS: Mid secretory
P: Proliferative
PC: Principal component
SMR: Summary-data-based Mendelian randomisation
TC: Total cholesterol
TG: Triglycerides
TSS: Transcription start site
