# Supplementary material for: Genetic regulation of methylation in human endometrium and blood and gene targets for reproductive diseases
Source: Clin Epigenetics. 2019 Mar 14;11:49. doi: 10.1186/s13148-019-0648-7 (PMC6416889; doi:10.1186/s13148-019-0648-7)
Supplement: Supplementary file 1 — Supplementary Notes. (PDF 65 kb) [file 13148_2019_648_MOESM1_ESM.pdf]

Supplementary Notes

# Genetic regulation of methylation in human endometrium and blood and gene targets for reproductive diseases

Sally Mortlock [1], Restuadi Restuadi [1], Rupert Levien [1], Jane E. Girling [2,3], Sarah J. Holdsworth-Carson [2], Martin Healey [2], Zhihong Zhu [1], Ting Qi [1], Yang Wu [1], Samuel W. Lukowski [1], Peter A.W. Rogers [2], Jian Yang [1], Allan F. McRae [1], Jenny N. Fung [1], and Grant W. Montgomery [1]

1. The Institute for Molecular Bioscience, The University of Queensland, Brisbane, QLD 4072, Australia
2. University of Melbourne Department of Obstetrics and Gynaecology, and Gynaecology Research Centre, Royal Women's Hospital, Parkville VIC 3052, Australia
3. Department of Anatomy, University of Otago, Dunedin, New Zealand

**Supplementary Note 1:**

Thirty-five per cent (155,675) of the DNAm probe sites in endometrial samples were hypomethylated ($\beta<0.2$) in at least 90% of samples irrespective of menstrual cycle stage or endometriosis status and 22% (97,145) of the DNAm probe sites were consistently hypermethylated ($\beta>0.8$) (Figure 1A). The proportions and distributions of hypomethylated and hypermethylated sites was similar in blood sample DNA, with 37% (161,747) of the DNAm probe sites in blood DNA consistently hypomethylated and 33% (146,090) of the DNAm probe sites consistently hypermethylated (Figure 1A).

Cpg islands were defined as regions >500bp in length with > 5% GC sequence and an expected/observed CpG ratio of >0.65, shores were defined as regions 2kb upstream/downstream from CpG islands, shelves as regions between 2 and 4 kb from CpG islands and finally all remaining regions were defined as open sea.

Hypomethylated sites were more common in CpG islands (65%) compared to open sea regions (9%) (Figure 1B). The opposite was observed for hypermethylated sites which were more common in open sea regions (54%) than CpG islands (12%) (Figure 1B). Approximately 65% of hypomethylated sites in blood were located in CpG islands, 10% where in open sea regions, and 55% of hypermethylated sites in blood were in open sea regions and only 10% were in CpG islands (Figure 1C).

**Supplementary Note 2:**

The majority of loci (58%) from the M2T analysis show opposite directions of effect. For alleles increasing methylation at specific sites, gene expression decreases and vice versa. The remaining 42% of eQTL and mQTL associations had allelic effects in the same direction. Similarly, 65% of T2M associations had effects in the opposite direction.

**Supplementary Note 3:**

mQTL DNAm probes were also annotated to predicted regulatory regions, the majority were in promoters (27.7%), 15.5% in enhancers and 24% were in quiescent regions in endometrium, and similarly, 28.6% were in promoters, 15.5% in enhancers and 21.8% were in quiescent regions in blood.
