# Supplementary material for: Genetic regulation of methylation in human endometrium and blood and gene targets for reproductive diseases
Source: Clin Epigenetics. 2019 Mar 14;11:49. doi: 10.1186/s13148-019-0648-7 (PMC6416889; doi:10.1186/s13148-019-0648-7)
Supplement: Supplementary file 2 — Supplementary Figures. (PDF 4540 kb) [file 13148_2019_648_MOESM2_ESM.pdf]

Supplementary Figures

Genetic regulation of methylation in human endometrium and blood and gene targets for reproductive diseases

Sally Mortlock [1], Restuadi Restuadi [1], Rupert Levien [1], Jane E. Girling [2,3], Sarah J. Holdsworth-Carson [2], Martin Healey [2], Zhihong Zhu [1], Ting Qi [1], Yang Wu [1], Samuel W. Lukowski [1], Peter A.W. Rogers [2], Jian Yang [1], Allan F. McRae [1], Jenny N. Fung [1], and Grant W. Montgomery [1]

1. The Institute for Molecular Bioscience, The University of Queensland, Brisbane, QLD 4072, Australia
2. University of Melbourne Department of Obstetrics and Gynaecology, and Gynaecology Research Centre, Royal Women's Hospital, Parkville VIC 3052, Australia
3. Department of Anatomy, University of Otago, Dunedin, New Zealand

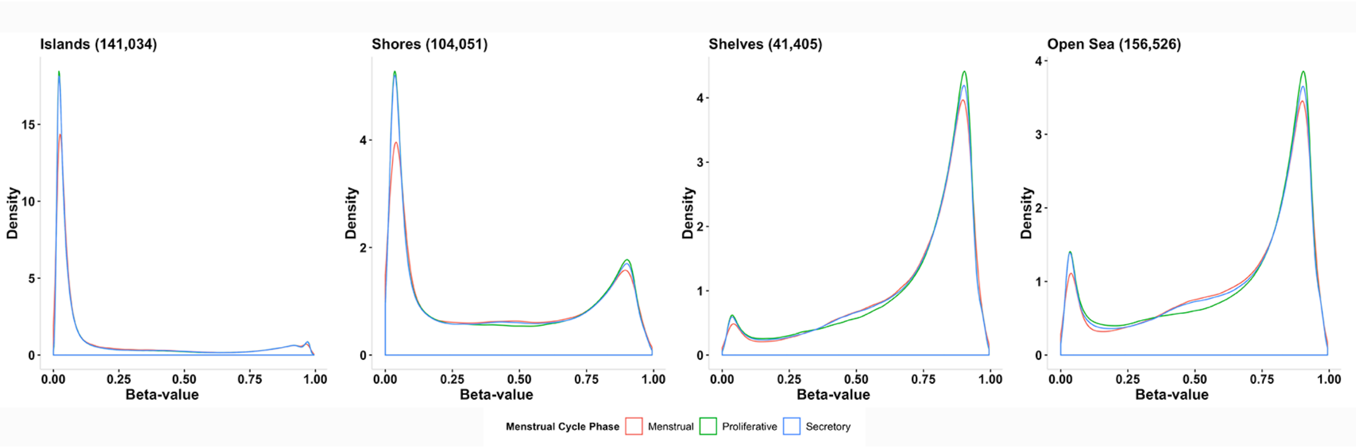

Figure S1. Density plots showing the distribution of beta values at each DNA methylation probe in CpG islands, shores, shelves and open sea regions in the menstrual, proliferative and secretory phases of the menstrual cycle in endometrium.

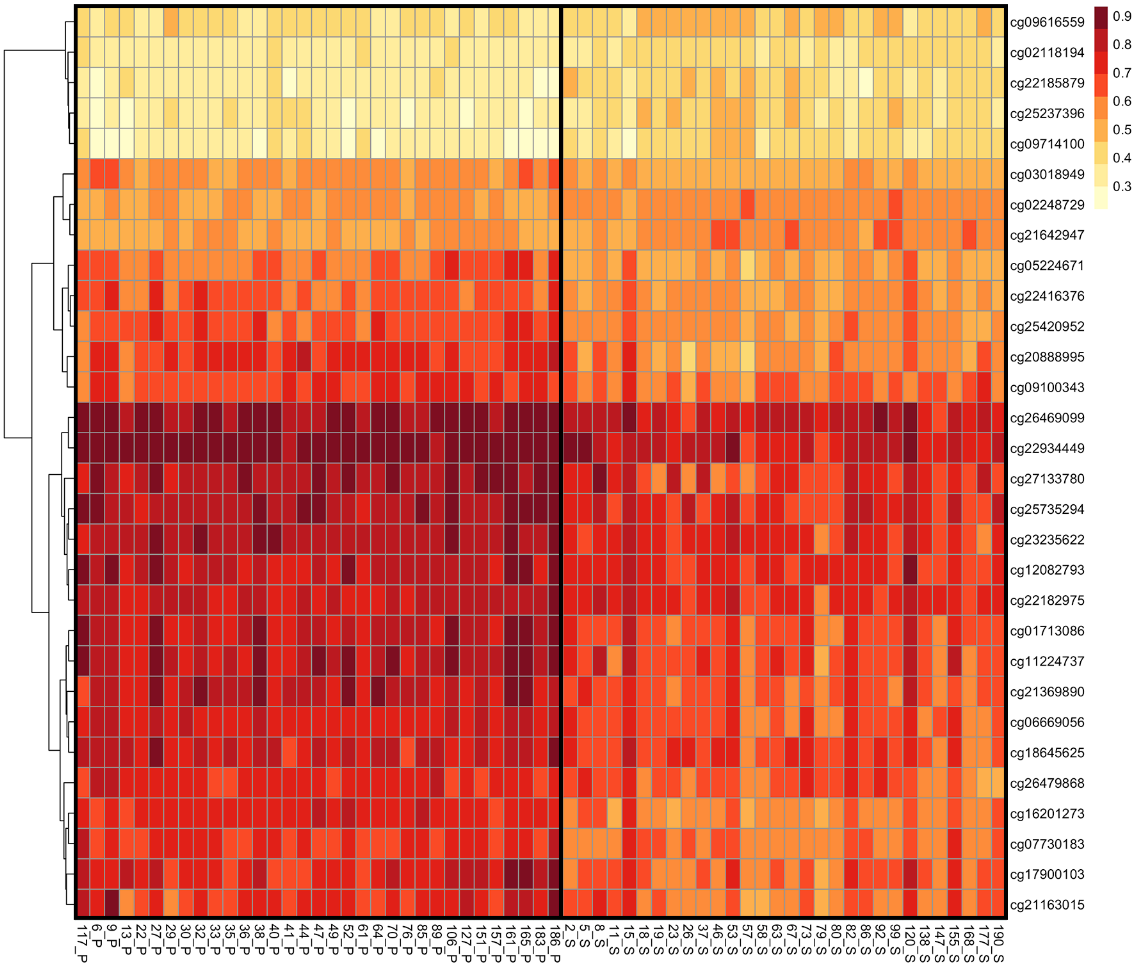

Figure S2. Heatmap showing the methylation profile of the top 30 most significantly differentially methylated DNAm probe sites between the Proliferative (P) and Secretory (S) phase. The two menstrual cycle stages are outlined in black. The colour key on the right corresponds to the beta value.

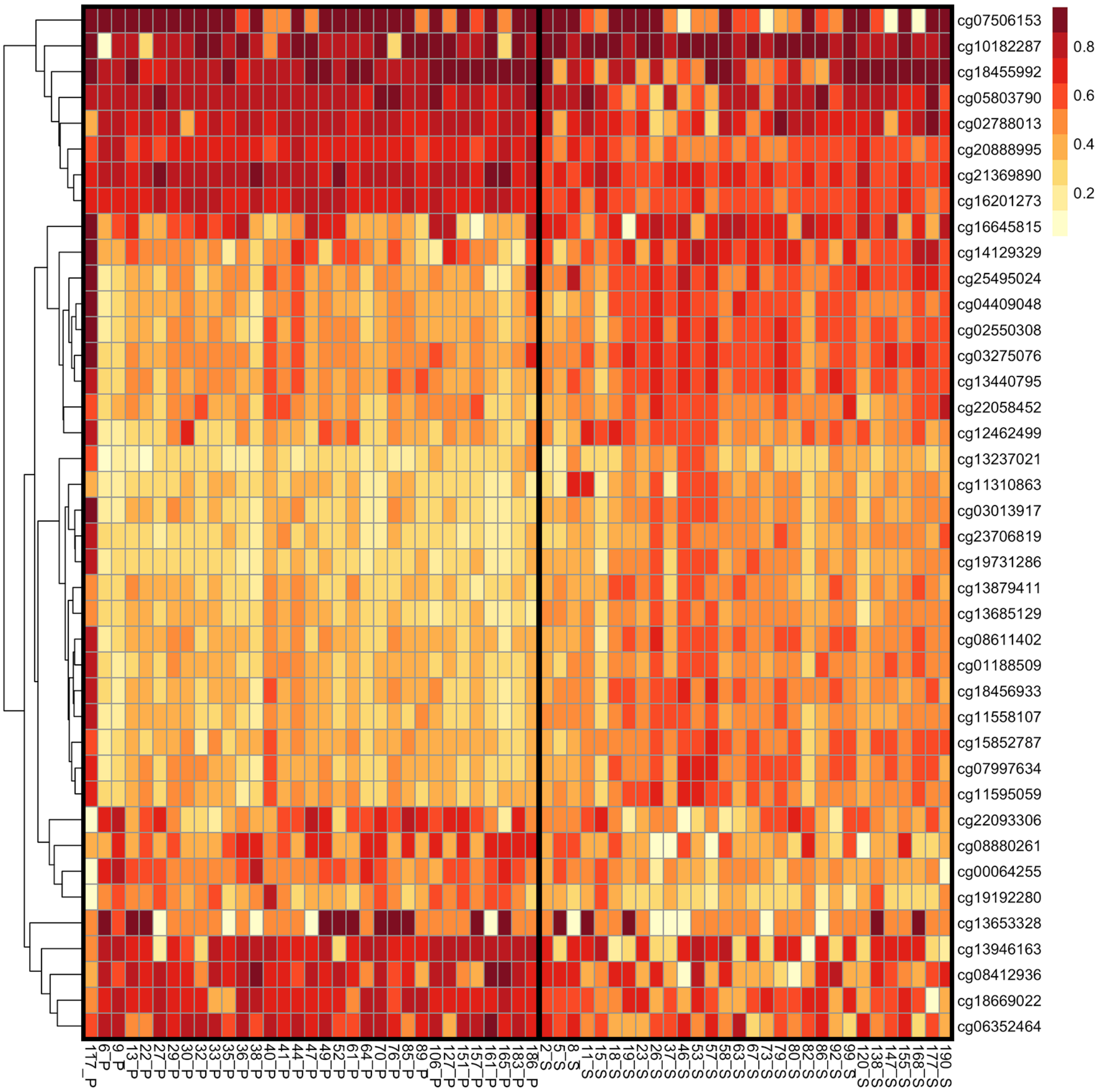

Figure S3. Heatmap showing the methylation profile of 40 DNAm probe sites with the biggest fold change between the Proliferative (P) and Secretory (S) phase. The two menstrual cycle stages are outlined in black. The colour key on the right corresponds to the beta value.

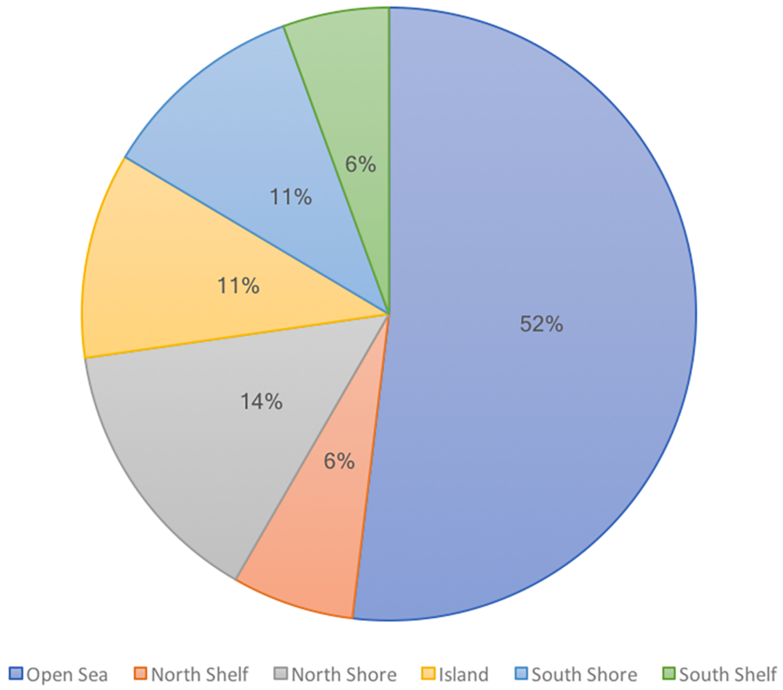

Figure S4. Proportion of differentially methylated CpG sites between proliferative (P) and Secretory (S) phases of the menstrual cycle located in CpG islands, shores, shelves and in open sea regions of the genome.

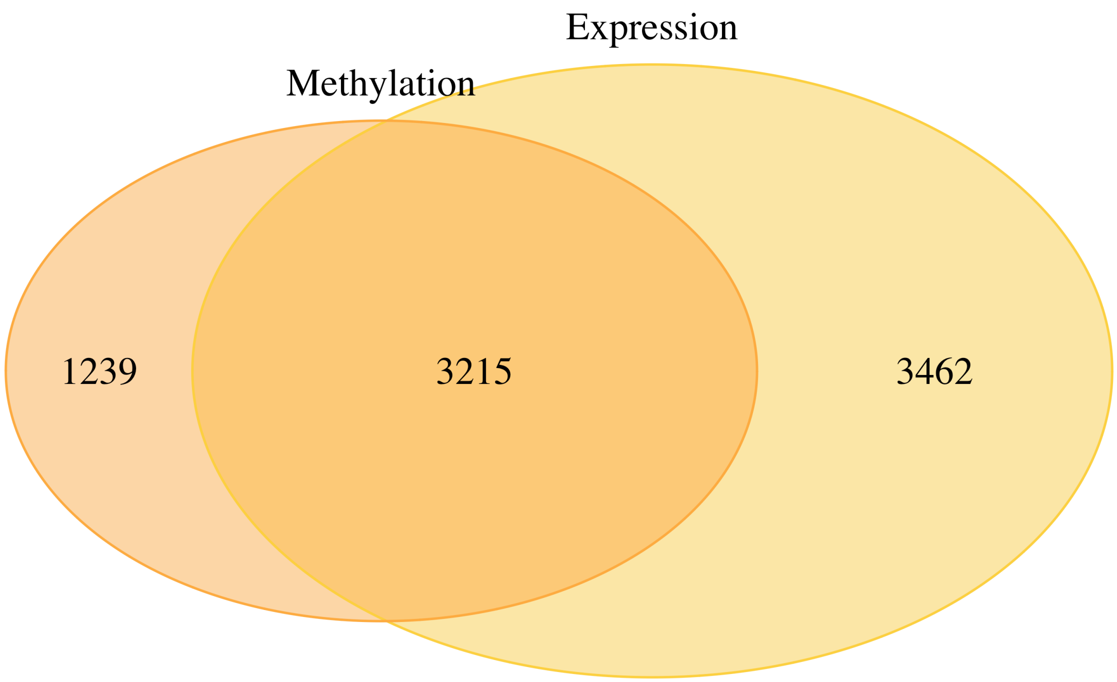

Figure S5. Venn diagram of overlap between genes differentially expressed (Expression, yellow) and methylated (Methylation, orange) between the proliferative and secretory phase of the menstrual cycle.

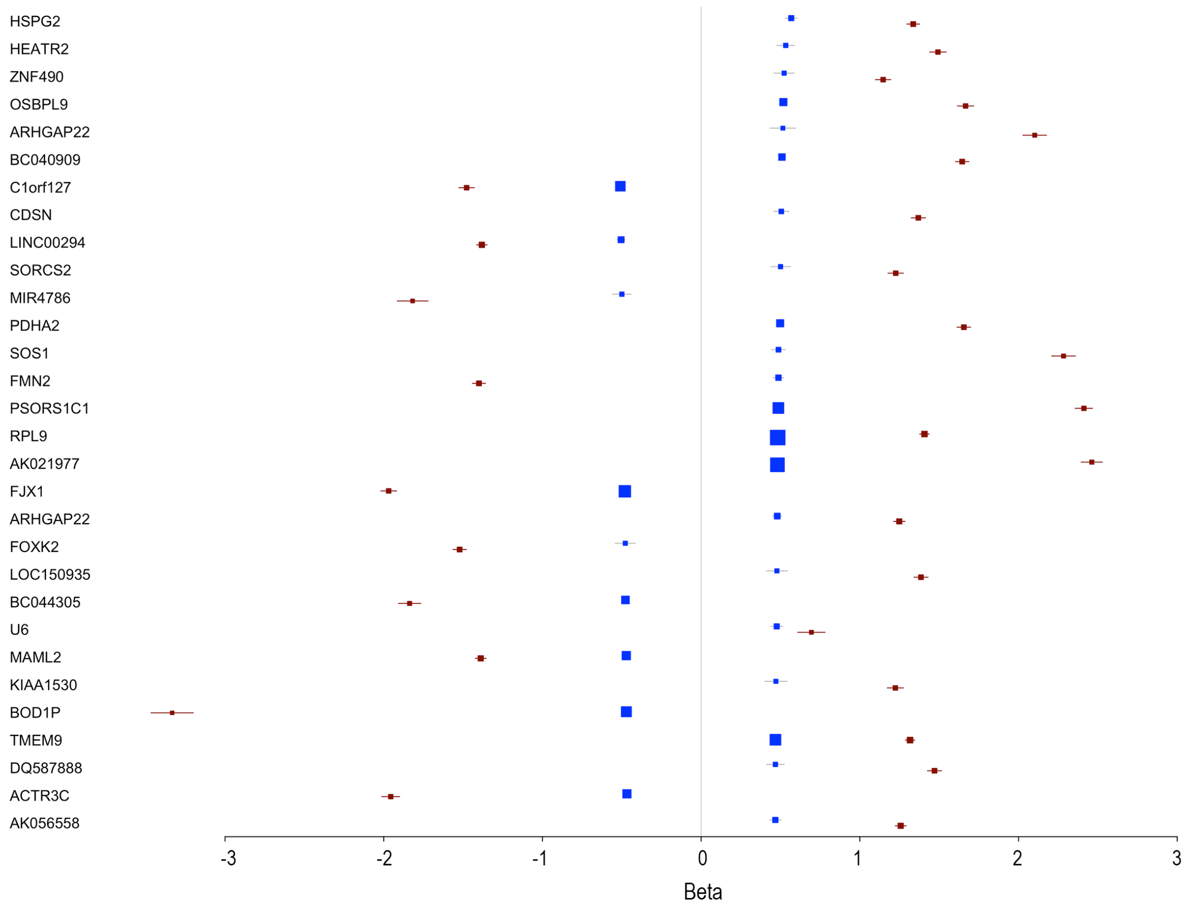

Figure S6. Effect size of the top 30 endometrial *cis*-mQTLs of largest effect (blue) compared with effect sizes published in blood (red).

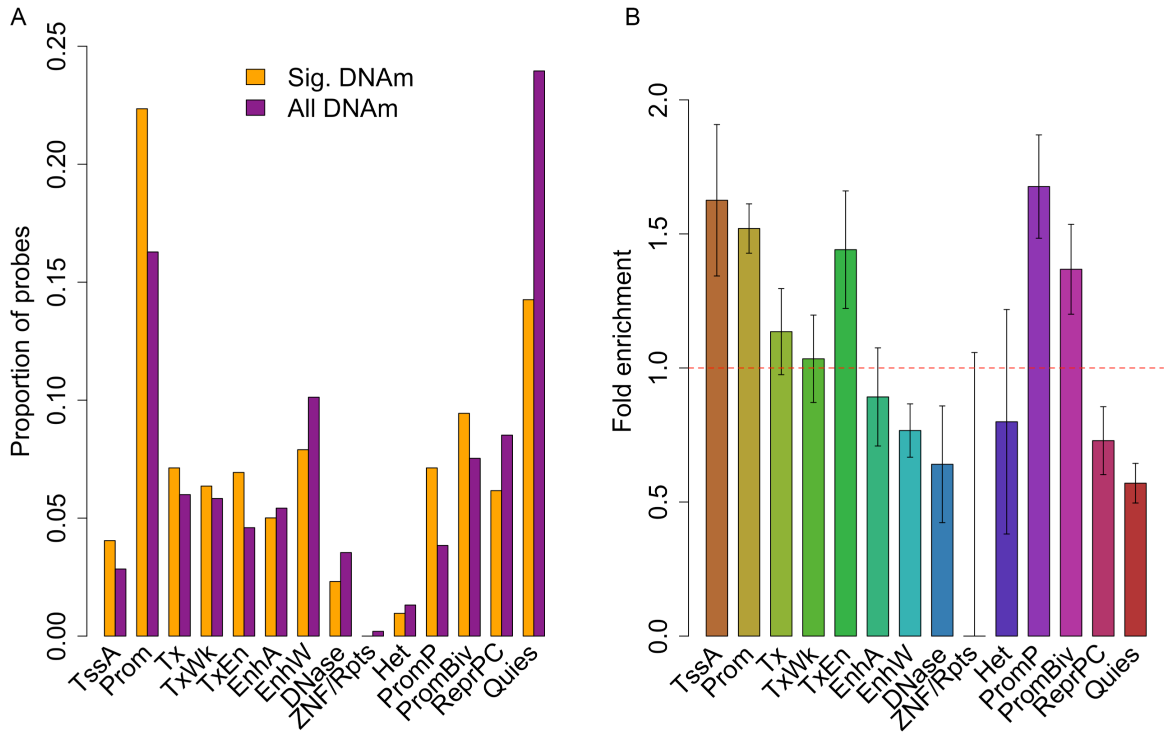

Figure S7. Enrichment of DNAm probes in 14 functional categories. A) Proportion of DNAm probes in each functional category for both transcript-associated DNAm probes (from M2T analysis) (orange) and all DNAm probes (purple). B) Fold change of significant DNAm probes when compared to probes sampled at random 500 times with matching DNAm variance. Error bars show the standard error calculated from the 500 random samples. Functional categories include active transcription start site (TssA), upstream/downstream TSS promoter (Prom), actively transcribed state (Tx), weak transcription (TxWk), transcribed and regulatory Prom/Enh (TxEn), active enhancer (EnhA), weak enhancer (EnhW), primary DNase (DNase), state associated with zinc-finger (ZNF/Rpts) protein genes, constitutive heterochromatin (Het), Poised promoter (PromP), bivalent regulatory states (PromBiv), repressed Polycomb states (ReprPC) and a quiescent state (Quies) as defined in Wu et.al (6).

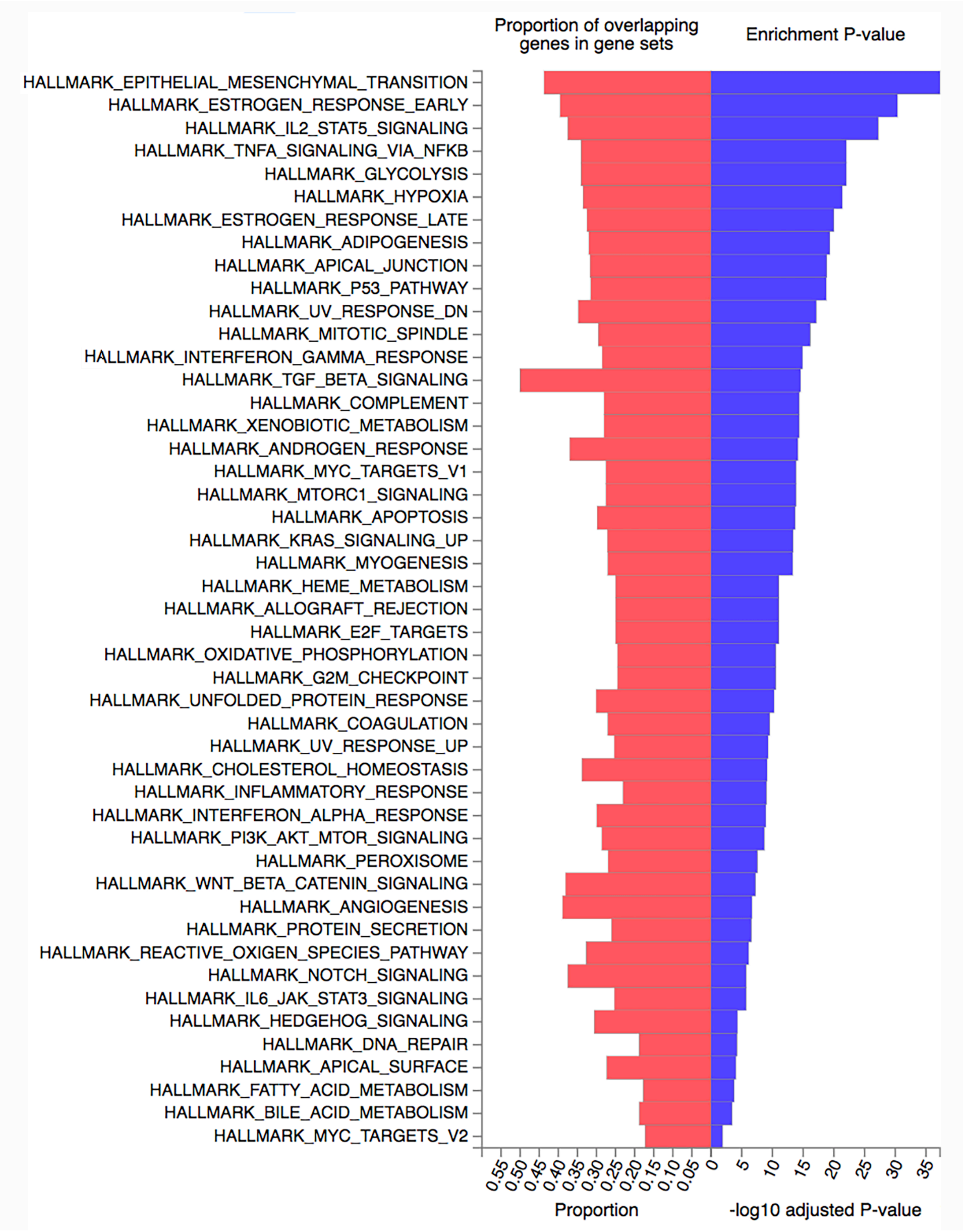

Figure S8. Hallmark pathways significantly enriched for genes that are both differentially methylated and expressed or not expressed in endometrium between phases of the menstrual cycle.

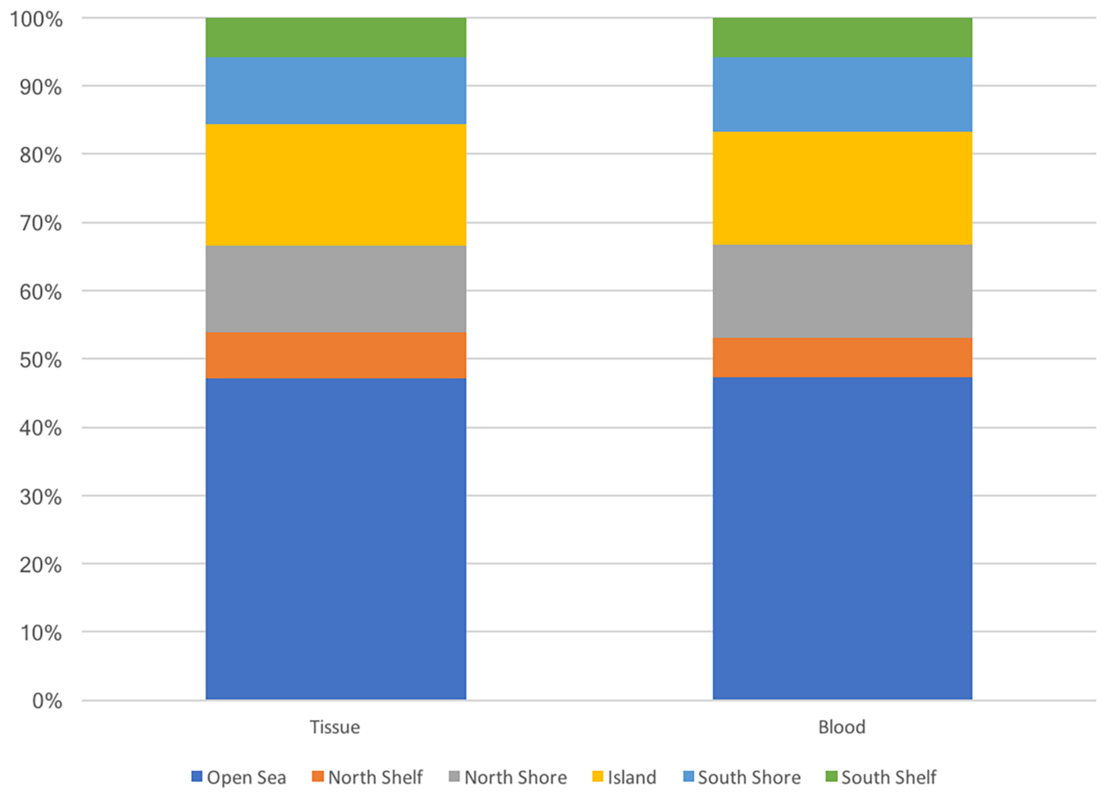

Figure S9. Proportion of endometrium and blood *cis*-mQTL CpG sites located in CpG islands, shores, shelves and in open sea regions of the genome.

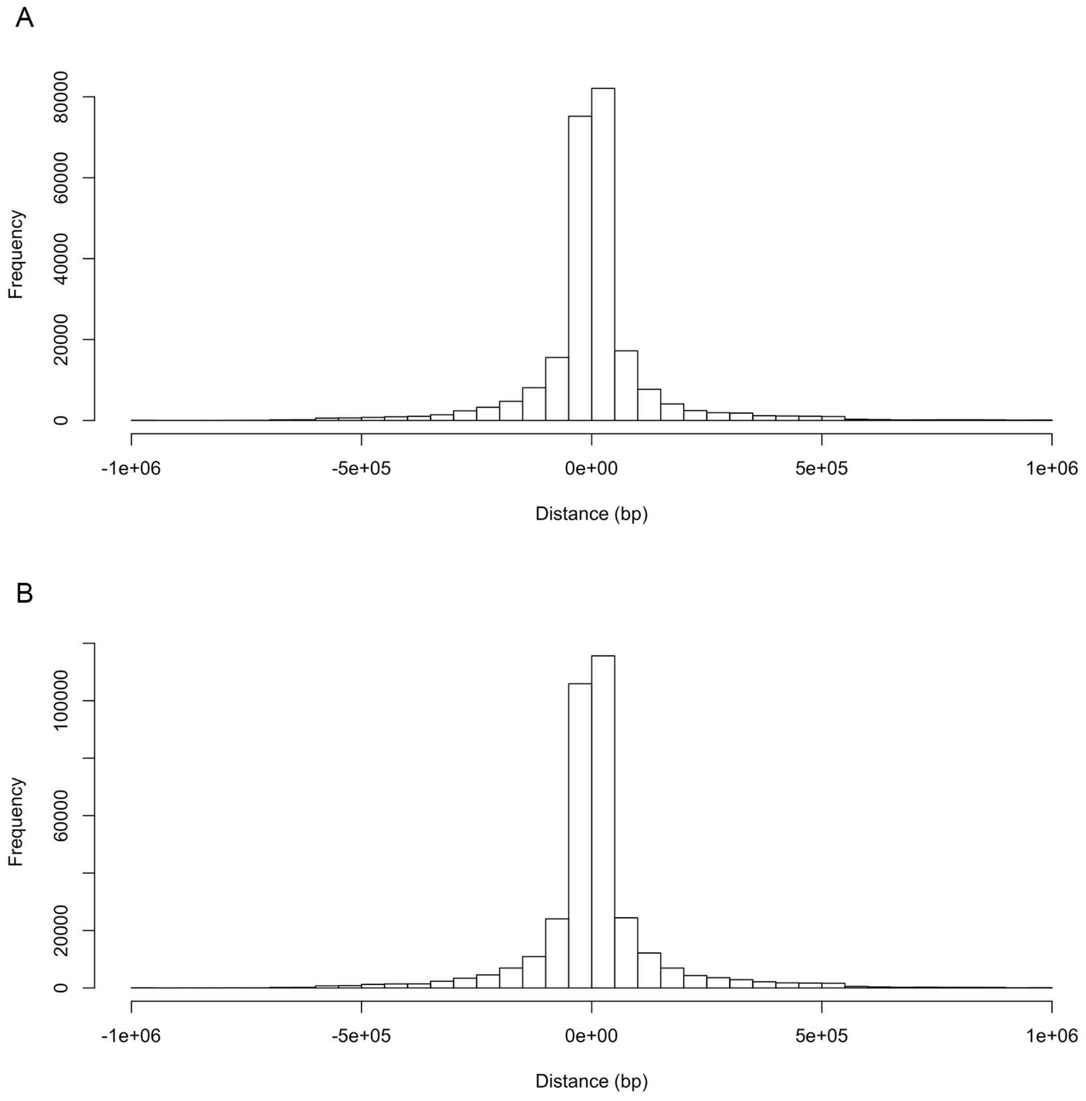

Figure S10. A) Distribution of the distance between significant mQTL SNPs and the DNAm probe site for which they are associated in endometrium. B) Distribution of the distance between significant mQTL SNPs and the DNAm probe site for which they are associated in blood.
